# Electrolyte disorders in the critically ill: a retrospective analysis

**DOI:** 10.1038/s41598-025-98677-7

**Published:** 2025-04-22

**Authors:** Kaspar Felix Bachmann, Benjamin Hess, Merli Koitmäe, Andreas Bloch, Adrian Regli, Annika Reintam Blaser

**Affiliations:** 1 Department of Intensive Care Medicine, Lucerne Cantonal Hospital, Lucerne, Switzerland; 2 Department of Anaesthesiology and Intensive Care, University of Tartu, Tartu, Estonia; 3 Department of Intensive Care Medicine, Bern University Hospital, Inselspital, University of Bern, Bern, Switzerland; 4 Estonian Genome Center, Institute of Genomics, University of Tartu, Tartu, Estonia; 5 Institute of Mathematics and Statistics, University of Tartu, Tartu, Estonia; 6 Department of Intensive Care, Fiona Stanley Hospital, Perth, WA Australia; 7 Medical School, The Notre Dame University, Fremantle, WA Australia; 8 Medical School, The University of Western Australia, Perth, WA Australia

**Keywords:** Electrolyte imbalance, Electrolyte disorder, Critical illness, Electrolyte substitution, Intensive care units, Endocrine system and metabolic diseases, Metabolic disorders

## Abstract

Several electrolyte disorders have been associated with adverse outcomes, but data on the coincidence of multiple disorders, interactions between different electrolytes and the impact of substitution on electrolyte levels are scarce. This study aimed to describe electrolyte disorders’ prevalence, incidence, and interaction in a consecutive cohort of ICU patients. Single-center retrospective study, including 2,056 consecutive adult ICU patients. Patients without laboratory data, those declining participation, and those receiving renal replacement therapy were excluded. Electrolyte levels and intravenous electrolyte administration were analyzed during the first 96 h after ICU admission using descriptive statistics, regression models and AUC-ROC analysis. Of 2392 admitted patients, 2056 were included. On admission, 312 patients (15.2%) had no electrolytes measured, 316 (15.4%) did not have any electrolyte disorder, 643 (31.3%) had one disorder, and 785 (38.2%) had multiple disorders. The most common electrolyte disorders on admission were hyperchloremia (56.0%, 977/1,744), hyperkalemia (18.7%, 326/1,744), hypophosphatemia (16.8%, 293/1,744), and hyperphosphatemia (16.4%, 286/1,744). Most patients (77.4%, 1,592/2,056) developed at least one new disorder during their ICU stay. A considerable number of patients (19.7%, 344/1,744) experienced electrolyte disorders in both directions (‘hypo’ and ‘hyper’) during their ICU stay. Linear regression models revealed that changes in one electrolyte level are often associated with alterations in other electrolyte levels. Most patients (99.2% 2,039/2,056) received intravenous electrolytes. Electrolyte overcorrection occurred in 89/365 patients (24.4%) for hypokalemia and 50/575 patients (8.7%) for hypophosphatemia. AUC-ROC analysis revealed cut-off points with the highest sensitivity and specificity at 30 mmol of potassium within 6 h and 45 mmol of phosphate within 15 h. Electrolyte disorders are highly prevalent in ICU patients, with significant co-incidence and interplay between these disorders. Knowledge regarding overcorrection risks may inform safe electrolyte administration protocols.

**Supplementary Information:**

The online version contains supplementary material available at 10.1038/s41598-025-98677-7.

## Introduction

Electrolyte disorders are common in patients admitted to the intensive care unit (ICU) and are linked with disease severity and adverse outcomes^1,2^.

Deviations of sodium and potassium predict worse clinical outcomes in critically ill patients and patients with acute kidney injury, COVID-19 infection, myocardial infarction, and trauma^3–8^. Magnesium disturbances are equally associated with disease severity and mortality^9,10^, while abnormal chloride levels are coupled with worse outcomes, particularly after neurosurgical procedures^11–13^. Extreme changes in calcium concentrations are also independently linked to increased mortality^14^.

Although correction of electrolyte disorders is part of daily ICU routine, the impact of this approach, including the correction rate, on patient-centered outcomes, remains unclear^15,16^. While hypophosphatemia and the associated refeeding syndrome may be deleterious^17,18^, optimal phosphate correction thresholds are unknown^19,20^. Hypophosphatemia outside of refeeding syndrome probably has a smaller patient impact, whereas hyperphosphatemia indicates disease severity^21–23^. The rate of sodium correction can affect mortality^24^, whereas calcium substitution in septic patients is not beneficial and may even cause harm^25^. Critically ill patients may benefit from a stable potassium level, but clear target ranges remain unknown^26^. Evidence-based indications, target levels, optimal dose, and speed to correct low electrolyte levels without causing overcorrection are unknown. Electrolyte disorders frequently affect multiple electrolytes simultaneously and interactions between electrolyte levels when substituting electrolytes are poorly examined.

In this study, we aimed to describe three key aspects of electrolyte disorders in critically ill patients: First, to establish the epidemiology by determining the prevalence of concurrent electrolyte disorders in ICU patients and documenting the incidence of new disorders during ICU stay. Second, to analyze patterns of electrolyte correction, focusing on substitution practices and identifying potential thresholds for overcorrection. Third, to explore the interplay between different electrolytes using regression analyses to identify potential associations between changes in electrolyte levels, which could inform more comprehensive and personalized approaches to electrolyte management. As full panel electrolyte measurements are part of standard care at the Department of Intensive Care Medicine in Lucerne, a dataset of consecutive patients admitted to the ICU allows to study these research questions. The relationship between electrolyte disorders and clinical outcomes will be addressed in subsequent analyses.

## Methods

After approval from the human ethical research committee (Ethikkomission Nordwest- und Zentralschweiz (EKNZ), project ID 2021 − 00334), we retrospectively collected data from all consecutive ICU patients between November 1st 2019 and December 31st 2020. The need for informed consent was waived by the ethic committee of Ethikkomission Nordwest- und Zentralschweiz due to the retrospective nature of this study. Presumed consent with an active withdrawal option for all ethics committee-approved retrospective studies is the standard practice at the study site. The study was performed in accordance with relevant local guidelines and the Declaration of Helsinki. The ICU is the sole provider of intensive care medicine in the Luzerner Kantonsspital (LUKS), a tertiary care center in the central region of Switzerland registering 51’801 hospital admissions during the study period.

The data was extracted from the clinical information system (LuKis, EPIC, Verona, WI, USA) via SQL, and a data analyst encrypted patient data. We included all patients aged *≥* 18 years. We excluded patients without laboratory data, those who actively declined general participation in retrospective research and those requiring intermittent or continuous hemodialysis. For patients with multiple ICU admissions during the study period, only data from their first admission were analyzed to ensure independent observations and avoid potential confounding from previous electrolyte and fluid management on the ward and during their first ICU stay.

### Study period

The maximum study period for each patient was 96 h (4 days, day 0 to 3) to balance data collection in the presence of the high turnover rate in our ICU population. Each study day was defined as starting at 7 am and ending at 06:59 am the following day. The duration of day 0 varied depending on admission time. Subsequent study days (days 1 to 3) consisted of 24 h each unless the patient died or was discharged during that day. Laboratory data collected within 6 h before ICU admission, including data from the ward, operating theatre, or emergency department, were included in the analysis.

### Data collection

The following patient data were extracted: primary diagnosis, age, gender, weight, ICU and hospital length of stay (LOS), and in-hospital mortality. From the laboratory database, we retrieved values for sodium, potassium, chloride, magnesium, phosphate, ionized calcium, base excess, pH, pO2, pCO2, lactate, albumin, glucose, hemoglobin, blood urea nitrogen (BUN), oxygen saturation, creatinine, and bilirubin. We also extracted data on intravenous (IV) fluid and intravenous electrolyte administration, vasopressor therapy, respiratory parameters, and Glasgow Coma Scale scores. This collected data allowed us to calculate Simplified Acute Physiology Score (SAPS II) and daily Sequential Organ Failure Assessment (SOFA) scores, including organ-specific failure sub-scores.

### Objectives and definitions

The primary objective of this study was to describe the prevalence and incidence of each electrolyte disorder in a mixed ICU cohort during a 4-day observation period. A secondary objective was to explore the relationship between different electrolyte disorders on admission and during the study period. Additionally, we analyzed the impact of electrolyte and fluid substitution on correction and overcorrection in the context of electrolyte disorders.

To evaluate the interactions between electrolyte changes over time, we included patients with at least two complete sets of electrolyte panels. Since the frequency of electrolyte measurements varied among patients, we based our analysis on the time interval corresponding to the least frequently measured electrolyte. We extracted the closest available value for every other electrolyte at each selected time point. As some electrolytes are measured as point of care (i.e. Sodium, Potassium, Ionized Calcium, Chloride) and some electrolytes are measured in the laboratory (i.e. Phosphate, Magnesium), there is a time difference in the time points. A visualization of this methodology is represented in supplementary Fig. 1. The electrolyte interplay was then assessed by calculating the changes in electrolyte levels within these predefined time windows. If data for all electrolytes were unavailable within a given period, that specific time interval was excluded from the analysis.

**Fig. 1:**
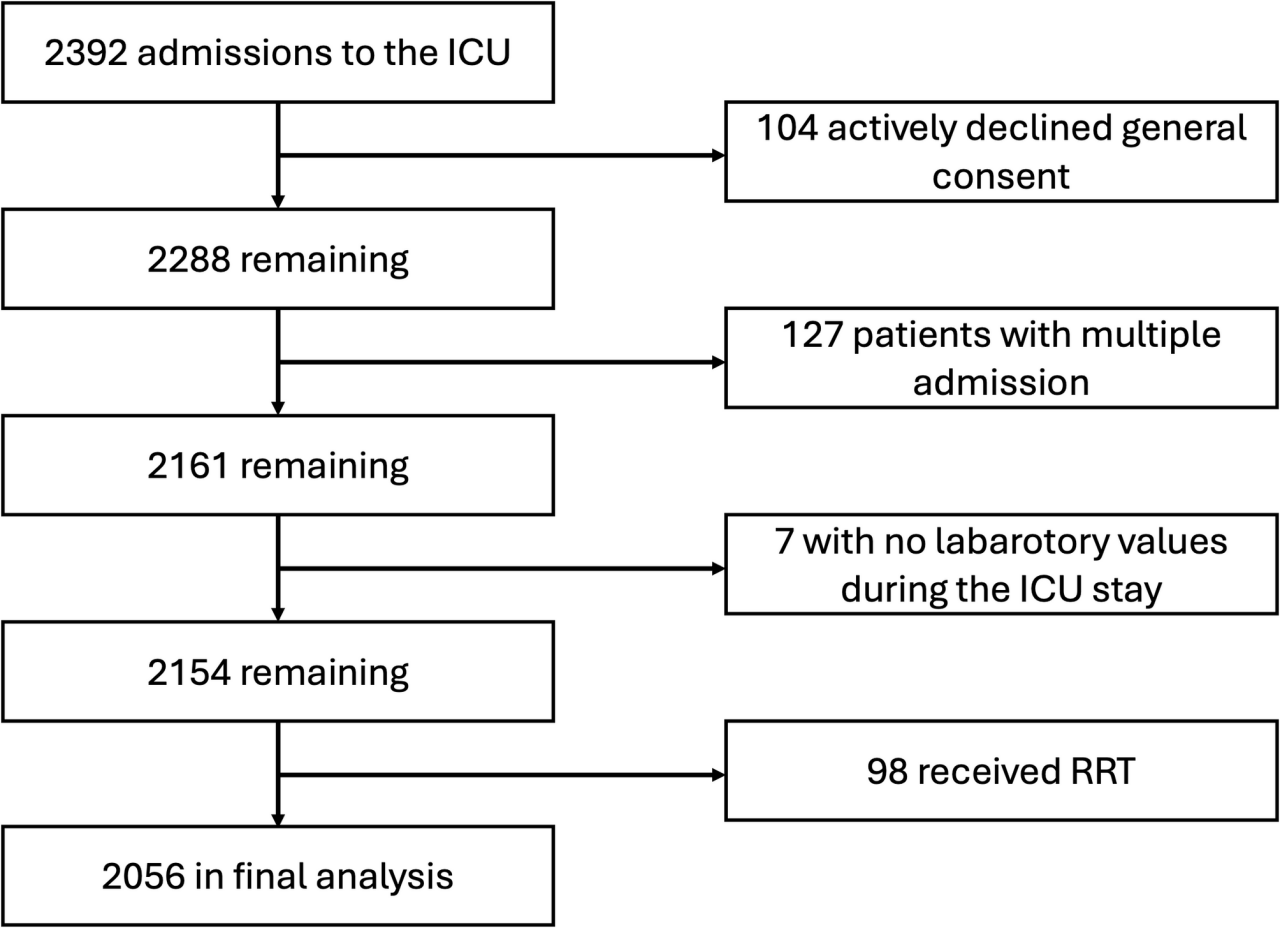
Flow chart showing the number of patients screened, excluded and included in the final dataset. Of the 2056 patients in the final analysis, only 1744 had electrolyte data upon admission.

Admission electrolyte values were taken from the first recorded electrolyte value during day 0 for each electrolyte. Concomitant electrolyte disturbances were assessed for admission electrolyte values. Electrolyte values were categorized into hypo, normal, or hyper based on reference ranges (see supplementary Table 1).

If patients were admitted with normal electrolyte values, we assessed whether they developed an electrolyte disorder during their ICU stay. We calculated the total amount of electrolyte administration provided for each patient and electrolyte. We also calculated the electrolyte difference between the lowest value and the value at the end of the study period or until correction or overcorrection occurred. Correction was defined as recording the first normal value after a hypo in patients who received electrolyte substitution, while overcorrection was defined as the first hyper after a hypo. Patients with hypo were categorized as uncorrected, corrected to normal values or overcorrection within the assessed study period. We assessed the correction and overcorrection of hypokalaemia and hypophosphatemia, as these two electrolyte deficiencies are frequently actively substituted based on a targeted electrolyte level. In contrast, we did not assess the correction of sodium and chloride levels as they are often modified with fluid restriction, administration of free or balanced fluids^27^. The severity of electrolyte disorders assessed for active correction was graded as follows^28^: Hypophosphatemia grade 1: < 0.87 and ≥ 0.65, grade 2: < 0.65 and ≥ 0.32, grade 3: < 0.32 and hypokalemia grade 1: < 3.4 and ≥ 3, grade 2: < 3 and ≥ 2.5, grade 3: < 2.5^20,29,30^.

### Statistical analysis

Data are presented as median with interquartile ranges or numbers with proportions. Heat maps, as well as parallel coordinate plots, were used to visualize data over time. Bivariable logistic regression models were used to test the associations between clinical data (e.g. age, mortality, etc.) and the electrolyte disorders.

To evaluate the co-occurrence of electrolyte disorders at admission, disorders were classified as present or absent without distinguishing between hypo- or hyper electrolyte states. Multivariable logistic regression models were used to assess the associations between changes in each electrolyte disorder (entered as a yes/no categorical variable), thus creating 6 models with each disorder as the dependent variable and all remaining disorders (e.g. five predictors) as independent variables.

Similarly, we used linear mixed-effects linear regression models to examine electrolyte interplay (i.e., association of changes between different electrolyte levels). We created six models, each with one electrolyte change as the dependent variable and the changes in the other five electrolytes as independent variables. Delta bicarbonate was added to each model to represent changes in acid-base balance, resulting in six independent variables for each model. Patients were entered as random effects to account for repeated measurements. We computed the partial dependence of each electrolyte in the models to visualize the impact on the dependent variable. We used adjusted R² values to evaluate the overall fit of each model. To visualize the electrolyte interplay, we created scatter plots of the raw data for each pair of electrolyte changes. We overlaid these plots with the partial dependence plots derived from the mixed-effects models to illustrate the predicted relationships while controlling for other variables in the model.

To assess the likelihood of electrolyte substitution leading to overcorrection, we used multivariable logistic regression models with overcorrection as the dependent variable and an interaction term of (“the grade of electrolyte disorder” times “the amount of substitution up to the maximum laboratory value”) as independent variables. We performed an AUC-ROC analysis for each grade of hypokalemia and hypophosphatemia to identify possible cut-off discriminating correction and overcorrection using the index of unity^31,32^. A p-value of < 0.05 was considered statistically significant. All data and statistical analyses were performed using MatLab (r2023a, Natwick, Massachusetts, US).

## Results

### Study population

We screened 2392 patients and 2056 were included (Fig. 1). The study population had a median age of 67 [55 to 77] years, and 68.9% were male. The median ICU and hospital LOS were 1.0 [0.8 to 2.0] and 9 [5 to 15] days, respectively. In-hospital mortality was 11.0% (227 patients). The median SAPS II was 31 [23 to 42]. Among the included patients, 49 (2.3%) were admitted for COVID-19 infection as a primary diagnosis, with a median ICU length of stay of 4.9 [1.5 to 8.2] days. Table 1 presents the demographics of the entire study population and the patients with different electrolyte disorders based on their admission values. Throughout the 96-hour study period, 1904 patients (92.6%) had at least one measurement of each studied electrolyte, while 462 patients (22.5%) remained in the ICU for the maximum study duration of 96 h.

**Table 1:** Patient characteristics for the total study population (N = 2056) and subgroups based on electrolyte disorders identified on admission.

	Total	Missing	No disorder	Hypo Na^+^	Hyper Na^+^	Hypo K^+^	Hyper K^+^	Hypo Cl^−^	Hyper Cl^−^	Hypo Mg^2+^	Hyper Mg^2+^	Hypo PO_4_^3^_−_	Hyper PO_4_^3−^	Hypo Ca^2+^	Hyper Ca^2+^
N	2056	312 (15.2%)	316 (15.4%)	268 (13.0%)	73 (3.6%)	170 (8.3%)	326 (15.9%)	108 (5.3%)	977 (47.5%)	98 (4.8%)	217 (10.6%)	293 (14.3%)	286 (13.9%)	136 (6.6%)	39 (1.9%)
Age	67.1 [55.2–76.7]	65.3 [51.2–78.3]	66.6 [54.4–76.8]	68.0 [56.4–77.3]	65.3 [47.8–74.7]	62.5 [52.0-75.8]#	70.0 [59.9–77.4]#	67.0 [57.4–75.7]	66.6 [54.4–76.5]	64.2 [52.4–76.6]	70.6 [60.5–78.3]#	68.5 [55.1–76.8]	70.6 [60.3–78.9]#	66.6 [57.7–77.6]	73.0 [65.7–81.6]§
Male, n (%)	1416 (68.9%)	225 (72.1%)	226 (71.5%),	162 (60.4%)	51 (69.9%)	87 (51.2%)#	247 (75.8%)#	60 (55.6%)#	677 (69.3%)	57 (58.2%)§	155 (71.4%)	227 (77.5%)#	183 (64.0%)	99 (72.8%)	22 (56.4%)
Weight, kg	75.1 [66.2–86.0]	74.1 [69.1–88.5]	77.6 [66.2–85.7]	70.2 [62.1–81.6]#	74.1 [68.9–81.2]	71.3 [64.2–81.3]#	75.1 [67.7–88.8]§	70.0 [62.2–81.0]#	75.9 [67.7–86.2]	70.0 [60.7–84.0]§	77.0 [66.0-87.4]	76.1 [69.1–86.0]	76.1 [65.8–87.9]	74.1 [67.7–87.6]	71.1 [62.5–80.3]
SAPS II	31 [23–42]	30 [21-42.5]	28 [21–36]#	36 [27–46]#	39 [25.8–51.3]#	34 [25–47]#	38 [29–49]#	36 [29.5–50]#	30 [22–41]§	32 [24–42]	34 [26–49]#	30 [23–41]	40 [30–52]#	40 [30.5–56]#	36 [28.3–46.3]
In hospital mortality, n (%)	227 (11.0%)	50 (16.0%)	23 (7.3%)	40 (14.9%)§	12 (16.4%)	30 (17.6%)#	53 (16.3%)#	22 (20.4%)#	91 (9.3%)§	9 (9.2%)	33 (15.2%)#	29 (9.9%)	56 (19.6%)#	23 (16.9%)§	7 (17.9%)
ICU LOS, days	1.0 [0.8-2.0]	0.9 [0.4–1.7]	1.0 [0.8–1.9]	1.2 [0.8–2.5]	1.2 [0.7–3.1]	1.2 [0.8–2.8]#	1.1 [0.8–2.3]	1.4 [0.8–2.7]	1.0 [0.8-2.0]	0.9 [0.8–1.9]	1.1 [0.9–2.8]	1.0 [0.8–2.2]	1.1 [0.8–2.7]	1.6 [0.9–4.3]#	1.0 [0.7–2.4]
Hospital LOS, days	9 [5–15]	5 [3–11]	9 [5–14]§	9 [5–16]§	7 [2-14.3]	8.5 [4–18]	11 [6–16]	8 [4-14.5]	10 [5–15]	9 [5–16]	11 [7–16]	7 [4–15]	12 [6–18]#	12 [6-16.5]	12 [4.5–20.8]
Fluids administered during ICU stay, L	1.7 [0.7–4.4]	0.8 [0.3–2.7]	1.4 [0.7–3.6]	2.0 [0.8–4.7]	2.1 [0.8–5.3]	1.9 [0.7–5.3]	2.1 [0.8–5.1]	2.3 [0.9–4.9]	1.8 [0.7–4.6]	1.4 [0.7–3.9]	4.1 [1.3–7.2]#	1.2 [0.5–3.8]§	2.3 [0.9–5.6]§	3.5 [1.4–7.3]#	1.6 [0.5–4.6]
Diagnosis Group: Cardiovascular	874 (42.5%)	116 (37.2%)	148 (46.8%)	74 (27.6%)	18 (24.7%)	48 (28.2%)	149 (45.7%)	23 (21.3%)	436 (44.6%)	33 (33.7%)	145 (66.8%)	141 (48.1%)	102 (35.7%)	60 (44.1%)	16 (41.0%)
Diagnosis Group: Sepsis	76 (3.7%)	11 (3.5%)	1 (0.3%)	35 (13.1%)	2 (2.7%)	16 (9.4%)	18 (5.5%)	11 (10.2%)	29 (3.0%)	6 (6.1%)	9 (4.1%)	16 (5.5%)	20 (7.0%)	8 (5.9%)	3 (7.7%)
Diagnosis Group: Respiratory	245 (11.9%)	42 (13.5%)	39 (12.3%)	44 (16.4%)	9 (12.3%)	20 (11.8%)	38 (11.7%)	20 (18.5%)	95 (9.7%)	8 (8.2%)	14 (6.5%)	35 (11.9%)	35 (12.2%)	18 (13.2%)	4 (10.3%)
Diagnosis Group: GI	163 (7.9%)	16 (5.1%)	12 (3.8%)	22 (8.2%)	4 (5.5%)	12 (7.1%)	37 (11.3%)	8 (7.4%)	91 (9.3%)	17 (17.3%)	10 (4.6%)	19 (6.5%)	46 (16.1%)	12 (8.8%)	6 (15.4%)
Diagnosis Group: Neurologic	322 (15.7%)	47 (15.1%)	61 (19.3%)	41 (15.3%)	12 (16.4%)	37 (21.8%)	25 (7.7%)	17 (15.7%)	143 (14.6%)	9 (9.2%)	21 (9.7%)	44 (15.0%)	35 (12.2%)	10 (7.4%)	5 (12.8%)
Diagnosis Group: Trauma	192 (9.3%)	42 (13.5%)	40 (12.7%)	13 (4.9%)	15 (20.5%)	15 (8.8%)	24 (7.4%)	4 (3.7%)	88 (9.0%)	7 (7.1%)	4 (1.8%)	19 (6.5%)	15 (5.2%)	14 (10.3%)	2 (5.1%)
Diagnosis Group: Metabolic / Endocrine	77 (3.7%)	23 (7.4%)	7 (2.2%)	24 (9.0%)	9 (12.3%)	13 (7.6%)	13 (4.0%)	23 (21.3%)	27 (2.8%)	8 (8.2%)	7 (3.2%)	16 (5.5%)	4 (1.4%)	8 (5.9%)	2 (5.1%)
Diagnosis Group: Hematologic	6 (0.3%)	1 (0.3%)	1 (0.3%)	0 (0.0%)	0 (0.0%)	0 (0.0%)	2 (0.6%)	0 (0.0%)	4 (0.4%)	0 (0.0%)	1 (0.5%)	0 (0.0%)	1 (0.3%)	0 (0.0%)	0 (0.0%)
Diagnosis Group: Genitourinary	45 (2.2%)	3 (1.0%)	3 (0.9%)	7 (2.6%)	3 (4.1%)	2 (1.2%)	10 (3.1%)	1 (0.9%)	30 (3.1%)	7 (7.1%)	1 (0.5%)	0 (0.0%)	18 (6.3%)	3 (2.2%)	1 (2.6%)
Diagnosis Group: Other	56 (2.7%)	11 (3.5%)	4 (1.3%)	8 (3.0%)	1 (1.4%)	7 (4.1%)	10 (3.1%)	1 (0.9%)	34 (3.5%)	3 (3.1%)	5 (2.3%)	3 (1.0%)	10 (3.5%)	3 (2.2%)	0 (0.0%)

Continuous variables are shown as median [interquartile range] and categorical variables as number (percentage). Percentages in the “N” row are relative to the total population, while other percentages are relative to each column’s subgroup. #: p-value < 0.01, §: p-value < 0.05 derived from logistic regression models (Supplementary table 2). The primary diagnosis of patients are shown as diagnostic groups. A more granular diagnosis list can be found in supplementary table 2. ICU: intensive care unit; LOS: length of stay; OR: odds ratio; SAPS II: simplified acute physiology score II. Na^+^: sodium. K^+^: potassium. Cl^−^: chloride. Mg^2+^: magnesium. PO_4_^3−^: phosphate. Ca^2+^: calcium.

### Prevalence of electrolyte disorders on admission

312 of 2056 patients (15.2%) had no electrolyte measurements upon ICU admission (i.e. no measurement available for day 0). Among the 1744 patients with admission electrolyte data, only 316 (18.1%) showed no electrolyte disorder. In these patients, the most common disorder was hyperchloremia (56.0%, 977/1744), followed by hyperkalemia (18.7%, 326/1744), hypophosphatemia (16.8%, 293/1744), and hyperphosphatemia (16.4%, 286/1744). Other notable disorders included hyponatremia (15.4%, 268/1744), hypomagnesemia (5.6%, 98/1744), and hypermagnesemia (12.4%, 217/1744). Hypercalcemia was the least common disorder (2.2%, 39/1744).

On admission, these electrolyte disorders were often concurrent. While 643 patients (31.3%) presented with only one disorder, 785 (38.2%) had two or more disorders. Multivariable logistic regression analysis revealed significant correlations between concurrent electrolyte disorders (Table 2). While chloride disorders were not strongly associated with other electrolyte disorders, sodium, potassium, phosphate, magnesium, and calcium disorders showed significant correlations with most other electrolyte disorders (Table 2).

**Table 2:** Multivariable regression models were used to evaluate the relationships between multiple electrolyte disorders present at admission.

Dependent Variable	Na^+^ disorder	Cl^−^ disorder	Mg^2+^ disorder	K^+^ disorder	PO_4_^3−^ disorder	Ca^2+^ disorder
Na^+^ disorder (*n* = 341) *		177 (51.9%), OR 0.84 [0.65 to 1.09]	64 (18.8%), OR 1.12 [0.81 to 1.55]	128 (37.5%), OR 2.01 [1.52 to 2.65]#	130 (38.1%), OR 1.62 [1.24 to 2.11]#	54 (15.8%), OR 1.89 [1.28 to 2.8]#
Cl^−^ disorder (*n* = 1085)	177 (16.3%), 0.84 [0.65 to 1.09]		165 (15.2%), OR 0.87 [0.68 to 1.12]	284 (26.2%), OR 1.18 [0.94 to 1.48]	317 (29.2%), OR 1.05 [0.86 to 1.29]	106 (9.8%), OR 1.34 [0.94 to 1.91]
Mg^2+^ disorder (*n* = 315)	64 (20.3%), 1.12 [0.81 to 1.55]	165 (52.4%), OR 0.87 [0.68 to 1.12]		117 (37.1%), OR 1.91 [1.46 to 2.51]#	118 (37.5%), OR 1.23 [0.95 to 1.6]	51 (16.2%), OR 2.11 [1.44 to 3.09]#
K^+^ disorder (*n* = 496)	128 (25.8%), 2.01 [1.52 to 2.65]#	284 (57.3%), OR 1.18 [0.94 to 1.47]	117 (23.6%), OR 1.91 [1.46 to 2.51]#		172 (34.7%), OR 1.34 [1.06 to 1.69]§	77 (15.5%), OR 2.21 [1.54 to 3.16]#
PO_4_^3−^ disorder (*n* = 579)	130 (22.5%), 1.62 [1.24 to 2.1]#	317 (54.7%), OR 1.05 [0.86 to 1.29]	118 (20.4%), OR 1.23 [0.95 to 1.6]	172 (29.7%), OR 1.34 [1.06 to 1.7]§		69 (11.9%), OR 1.63 [1.14 to 2.32]#
Ca^2+^ disorder (*n* = 175)	54 (30.9%), 1.9 [1.28 to 2.82]#	106 (60.6%), OR 1.32 [0.93 to 1.88]	51 (29.1%), OR 2.11 [1.44 to 3.09]#	77 (44%), OR 2.22 [1.55 to 3.18]#	69 (39.4%), OR 1.63 [1.15 to 2.32]#	

Hypo- and hyper- States for each electrolyte were combined into a single variable. For each combined disorder, the number and percentage of patients experiencing Both hypo- and hyper-states were calculated relative to the total number of patients with that disorder as the dependent variable. Each variable was entered once as the dependent variable and five times as independent variables. Odds ratios (OR) with 95% confidence intervals refer to the increased odds of two disorders being concurrent. #: p-value < 0.01, §: p-value < 0.05. Na^+^: sodium. K^+^: potassium. Cl^−^: chloride. Mg^2+^: magnesium. PO_4_^3−^: phosphate. Ca^2+^: Calcium. * n = total number of patients with disorder (incl. Both hypo and hyper).

In bivariable regression models, electrolyte disturbances were associated with indicators of disease severity (Table 1). Patients with at least one electrolyte disorder on admission had higher SAPS II scores (*p* < 0.01), higher in-hospital mortality (11.7% vs. 7.3%, *p* = 0.061), and longer hospital LOS (*p* < 0.05) compared to those without any disorder, although the signal was not significant for in-hospital mortality. Specifically, hyperkalemia was associated with higher age (OR 1.01 [1.01–1.02], *p* < 0.01), higher SAPS II scores (OR 1.03 [1.02–1.04], *p* < 0.01), and increased in-hospital mortality (OR 1.74 [1.24–2.43], *p* < 0.01). Hyperphosphatemia was associated with higher age (OR 1.02 [1.01–1.03], *p* < 0.01), higher SAPS II scores (OR 1.04 [1.03–1.04], *p* < 0.01), and increased in-hospital mortality (OR 2.61 [1.85–3.68], *p* < 0.01).

### Incidence of electrolyte disorders over the study period

**Fig. 2:**
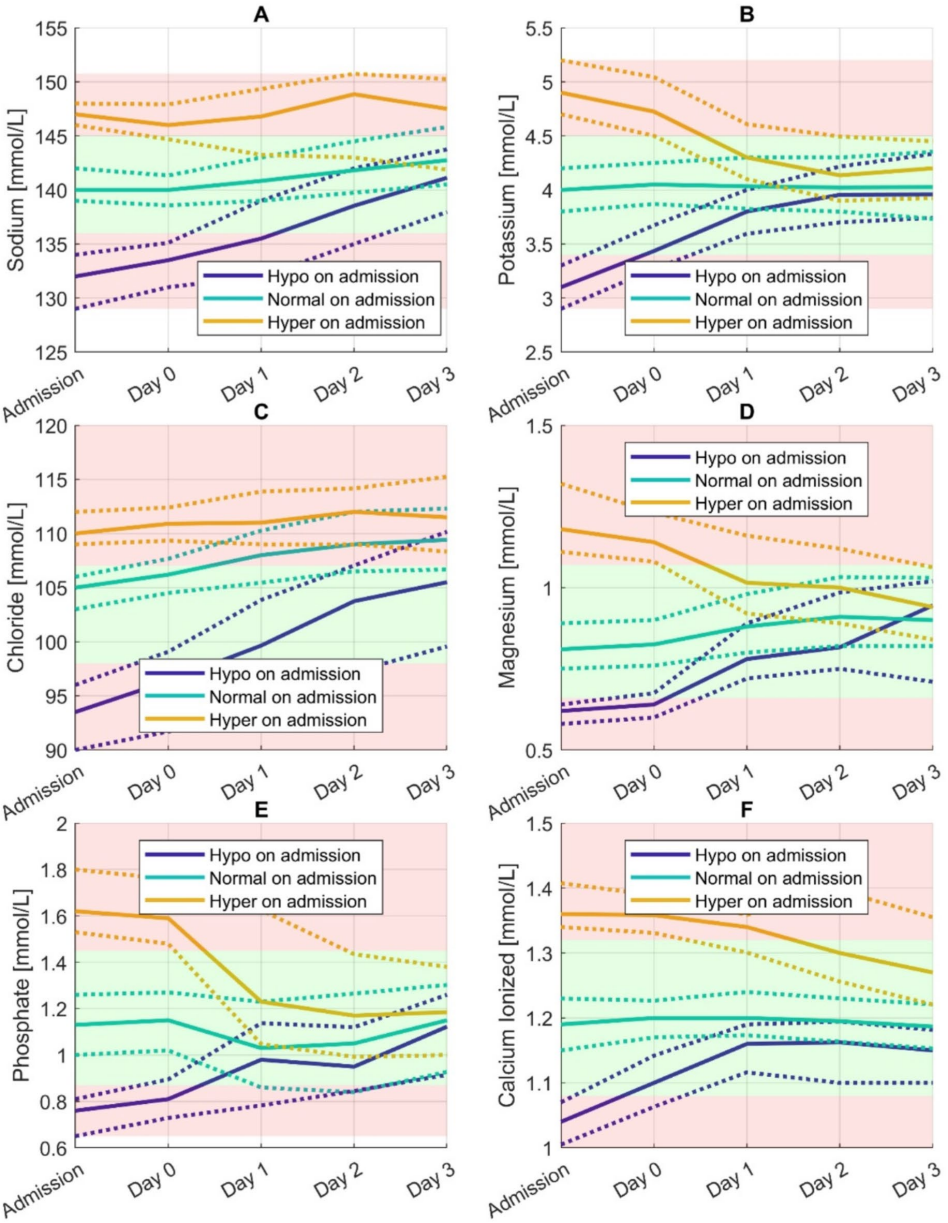
Parallel coordinate plots were used to visualize changes in electrolyte values (A to F) over time, with medians and interquartile ranges calculated for each electrolyte. Electrolyte disorders were categorized based on their status at admission. Green and red shaded areas indicate normal and abnormal values. Admission values represent the first values of day 0.

During the study period, most patients either presented with or developed at least one electrolyte disorder, with only 152/2056 patients (7.4%) remaining free of any electrolyte disorder throughout their ICU stay. Most electrolyte disorders found on admission normalized during the study period, except hyperchloremia (Fig. 2) with following correction frequency: hyponatremia 63.8% (171/268), hypernatremia 74.0% (54/73), hypokalemia 94.1% (160/170), hyperkalemia 81.3% (265/326), hypochloremia 75.0% (81/108), hyperchloremia 28.9% (282/977), hypomagnesemia 63.3% (62/98), hypermagnesemia 65.4% (142/217), hypophosphatemia 64.2% (188/293), hyperphosphatemia 54.2% (155/286), hypocalcemia 91.2% (124/136), and hypercalcemia 61.5% (24/39).

Many patients without the respective disorder on admission developed new electrolyte disorders during their ICU stay (Fig. 2 and Supplementary Fig. 2): hyponatremia 50.1% (269/537), hypernatremia 79.1% (276/349), hypokalemia 53.4% (195/365), hyperkalemia 62.7% (547/872), hypochloremia 24.5% (35/143), hyperchloremia 39.6% (640/1617), hypomagnesemia 19.0% (23/121), hypermagnesemia 44.2% (172/389), hypophosphatemia 49.0% (282/576), hyperphosphatemia 43.4% (219/505), hypocalcemia 54.4% (162/298) and hypercalcemia 77.3% (133/172).

During the entire study period, some patients experienced both hypo- and hypernatremia (2.8%, 58/2056), hypo- and hyperkalemia (6.0%, 124/2056), hypo- and hyperchloremia (2.3%, 47/2056), hypo- and hypermagnesemia (0.3%, 7/2056), hypo- and hyperphosphatemia (4.2%, 87/2056) and hypo- and hypercalcemia (1.0%, 21/2056).

### Electrolyte interplay

We analyzed 2,664 complete datasets of electrolyte changes from 1,398 patients. The median interval between consecutive full electrolyte panel measurements (i.e. start point to end point) was 20.3 h [12.0 to 24.0 h]. As a result, from point-of-care testing compared to standard laboratory testing, there was a small median time difference in the starting points of these intervals (34 min [0 to 47 min]) as well as for the endpoints (34 min [0 to 44 min], Supplementary Fig. 1).

**Fig. 3:**
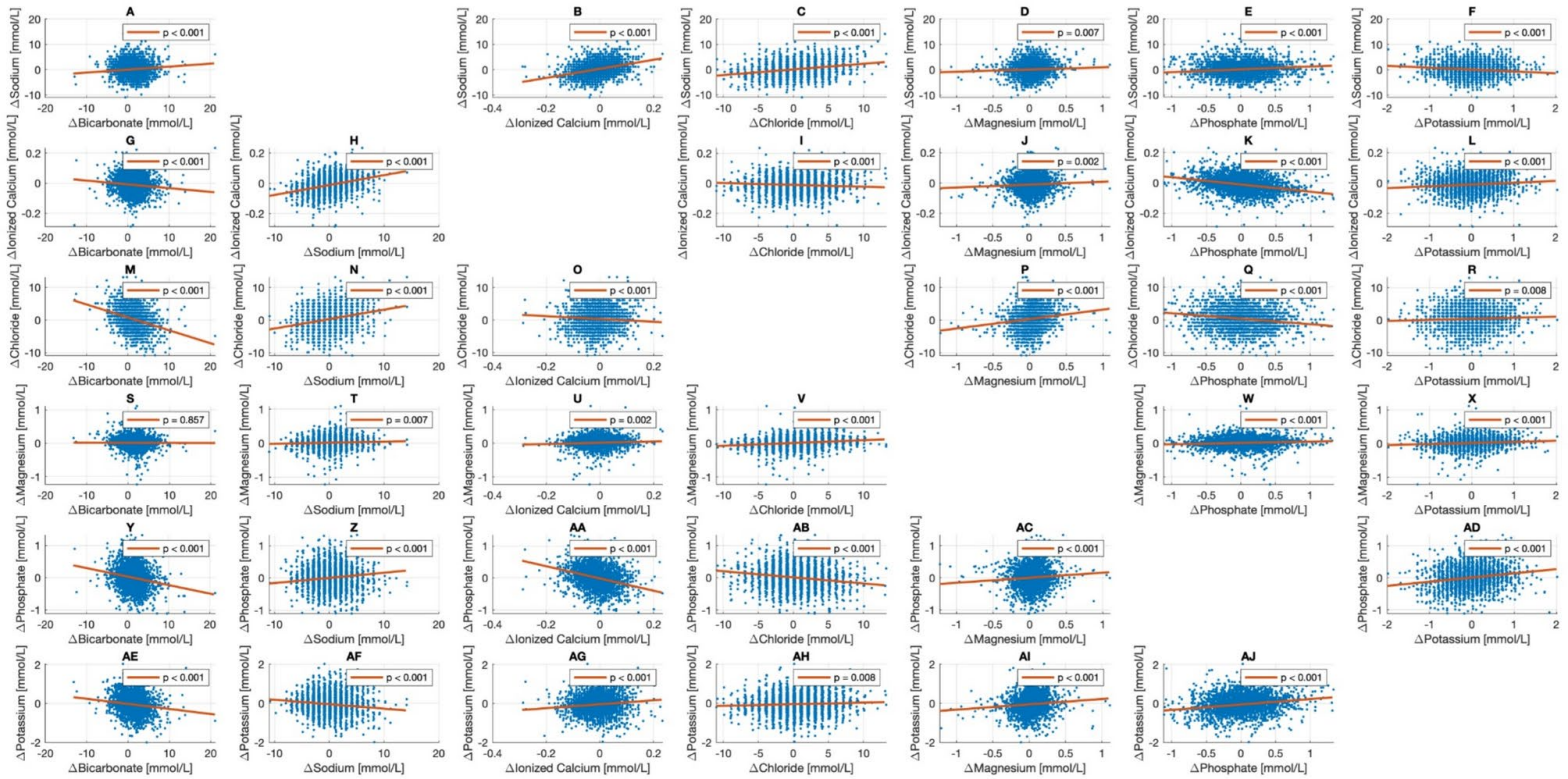
Interplay between different electrolyte changes. The delta refers to the change in the assessed time interval where data on changes of all electrolytes were available. The blue dots represent the actual data, the orange lines represent the partial dependence between the variables presented on the x-axis and the y-axis. The p-value is taken from each model referring to the corresponding variable on the x-axis. The figure presents the interactions for sodium (**A-F**), ionized calcium (**G-L**), chloride (**M-R**), magnesium (**S-X**), phosphate (**Y-AD**) and potassium (**AE-AJ**). The regression coefficients for each model can be found in Table 3.

Linear mixed-effects models adjusted for all electrolytes, including delta bicarbonate, showed highly significant associations between all variables (Table 3; Fig. 3). The magnitude of these associations is indicated by higher regression coefficients (Table 3, changes in mmol/L per 1 mmol/L) and are visualized in Fig. 3. For sodium changes (Fig. 3A-F), the strongest positive association was observed with changes in ionized calcium (regression coefficient 17.94 [16.06 to 19.82], *p* < 0.001), while changes in potassium showed a negative association (-0.73 [-0.95 to -0.52], *p* < 0.001). Changes in ionized calcium (Fig. 3G-L) were positively associated with changes in sodium (0.0065 [0.0058 to 0.0072], *p* < 0.001) and negatively associated with changes in phosphate (-0.048 [-0.054 to -0.042], *p* < 0.001). For chloride changes (Fig. 3M-R), a negative association was observed with bicarbonate changes (-0.40 [-0.44 to -0.35], *p* < 0.001), while a positive association was seen with sodium changes (0.29 [0.25 to 0.33], *p* < 0.001). Magnesium changes (Fig. 3S-X) showed positive associations with changes in chloride (0.0082 [0.0062 to 0.0103], *p* < 0.001) and phosphate (0.040 [0.021 to 0.059], *p* < 0.001). Phosphate changes (Fig. 3Y-AD) were negatively associated with changes in ionized calcium (-1.91 [-2.14 to -1.68], *p* < 0.001) and positively associated with changes in potassium (0.28 [0.22 to 0.33], *p* < 0.001). Potassium changes (Fig. 3AE-AJ) showed positive associations with changes in phosphate (0.28 [0.22 to 0.33], *p* < 0.001) and magnesium (0.27 [0.16 to 0.38], *p* < 0.001), and a negative association with changes in sodium (-0.02 [-0.03 to -0.02], *p* < 0.001). The models showed a weak fit for the data, indicated by low R² values (ranging from 0.06 for magnesium to 0.19 for sodium, chloride, and ionized calcium) and visualized by the scattered data in Fig. 3.

**Table 3:** Multivariable regression models were used to evaluate associations between concurrent electrolyte changes.

Dependent variable	ΔBicarbonate	ΔSodium	ΔIonized calcium	ΔChloride	ΔMagnesium	ΔPhosphate	ΔPotassium	Ajdusted *R*^2^
ΔSodium	0.12 [0.07 to 0.16]#		17.94 [16.06 to 19.82]#	0.22 [0.19 to 0.26]#	0.86 [0.24 to 1.49]#	1.1 [0.79 to 1.42]#	-0.73 [-0.95 to -0.52]#	0.19
ΔIonized calcium	-0.003 [-0.003 to -0.002]#	0.007 [0.006 to 0.007]#		-0.001 [-0.002 to -0.001]#	0.019 [0.007 to 0.031]#	-0.048 [-0.054 to -0.042]#	0.012 [0.008 to 0.016]#	0.19
ΔChloride	-0.4 [-0.44 to -0.35]#	0.29 [0.25 to 0.33]#	-4.34 [-6.61 to -2.07]#		2.9 [2.19 to 3.61]#	-1.68 [-2.04 to -1.33]#	0.33 [0.09 to 0.58]#	0.19
ΔMagnesium	-0.0002 [-0.003 to 0.002]	0.003 [0.001 to 0.005]#	0.192 [0.071 to 0.313]#	0.008 [0.006 to 0.01]#		0.04 [0.021 to 0.059]#	0.032 [0.019 to 0.045]#	0.06
ΔPhosphate	-0.03 [-0.03 to -0.02]#	0.02 [0.01 to 0.02]#	-1.91 [-2.14 to -1.68]#	-0.02 [-0.02 to -0.01]#	0.16 [0.08 to 0.23]#		0.13 [0.11 to 0.16]#	0.16
ΔPotassium	-0.03 [-0.03 to -0.02]#	-0.02 [-0.03 to -0.02]#	1.01 [0.66 to 1.36]#	0.01 [0.00 to 0.01]#	0.27 [0.16 to 0.38]#	0.28 [0.22 to 0.33]#		0.09

Regression coefficients with a 95% confidence interval are provided. The rows represent the dependent variables, while the columns represent the independent variables. All variables are measured in Mmol/L. The estimates from the linear mixed-effects regression models refer to the estimated increase or decrease in the dependent variable per change of 1 Mmol/L.in the independent variable. A visualization of the partial dependence of each model as well as the underlying data can be found in Fig. 3. #: p-value < 0.01.

### Risk of overcorrection through intravenous fluids and electrolytes

**Fig. 4:**
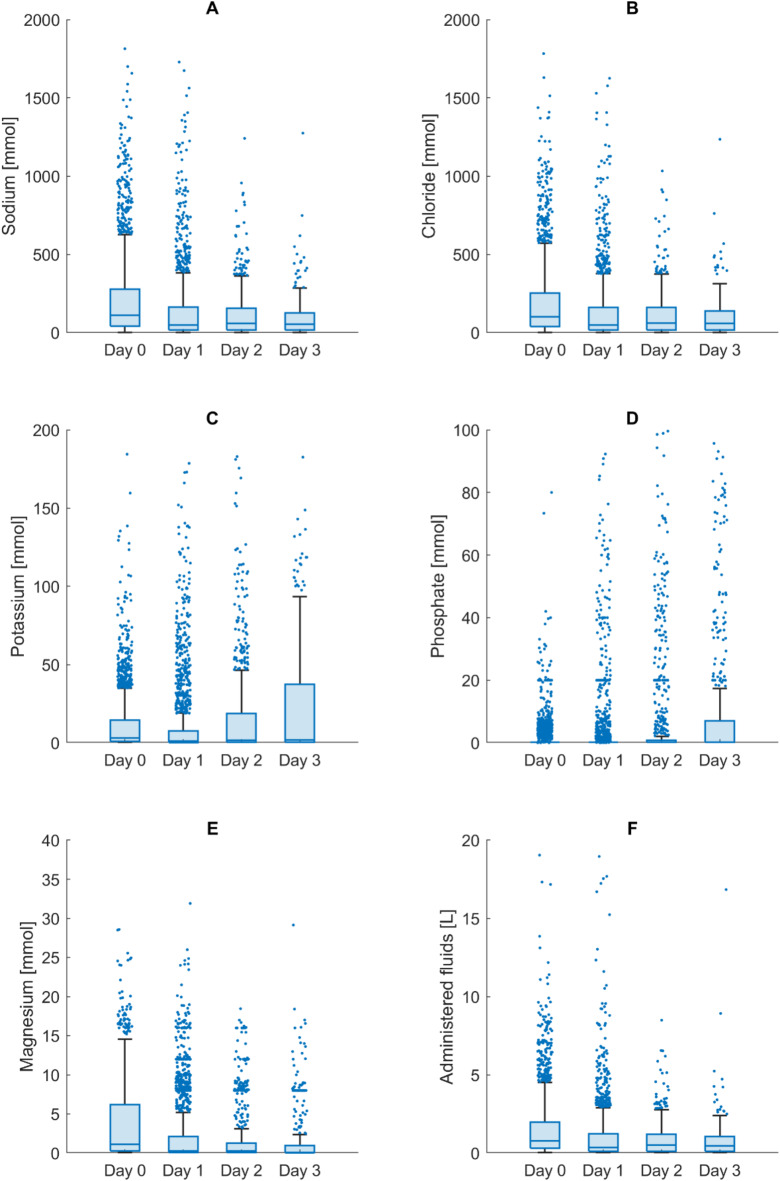
Amount of IV administration of electrolytes (mmol) and fluids (L) per study day. For improved visibility, the y-axis was adjusted according to the maximum values. **A**: Sodium substitution (y-axis range of 0 to 2000 mmol, maximum 7433 mmol). **B**: Chloride substitution (y-axis range of 0 to 2000 mmol, maximum 7291 mmol). **C**: Potassium substitution (y-axis range of 0 to 200 mmol/L, maximum 368 mmol). **D**: Phosphate substitution (y-axis range of 0 to 100 mmol, maximum 166 mmol). **E**: Magnesium substitution (y-axis range of 0 to 40 mmol, maximum 49 mmol). **F**: Intravenous fluids (y-axis range of 0 to 20 L, maximum 49.0 L).

Most patients received IV fluids and electrolytes during the study period. Sodium or chloride was administered to 2038 (99.1%) patients, with a median amount of 240 mmol [94 to 609] and 225 mmol [89 to 568], respectively. Potassium was administered to 1878 (91.3%) patients with a median of 10 mmol [3 to 43]. Phosphate was administered to 707 (34.4%) patients with a median amount of 9 mmol [6 to 40]. Magnesium was administered to 1928 (93.8%) patients with a median of 5 mmol [1 to 11]. IV fluids were administered to 2038 (99.1%) patients with a median volume of 1,723 ml [677 to 4,436]. The pattern of electrolyte administration varied over the study period (Fig. 4). Sodium chloride was administered mainly at the start of the study period as IV fluid therapy, while potassium and phosphate administration increased over the study period.

Of the 365 patients with hypokalemia on admission or during their ICU stay, 38 (10.4%) were not corrected to normal, 238 (65.2%) were corrected to normal values, and 89 (24.4%) were overcorrected. The median time between the recorded minimum potassium value and correction, overcorrection, or the end of the study period was 5.6 h [2.7 to 11.9]. Multivariable logistic regression analysis showed that for every 10 mmol of potassium administered, the odds of overcorrection increased by 18% for grade 1 hypokalemia (OR 1.19 [1.11 to 1.28], *p* < 0.001) and 18% for grade 2 hypokalemia (OR 1.18 [1.07 to 1.30], *p* < 0.001).

ROC analysis identified a cut-off for overcorrection of 30 mmol (sensitivity: 66.2%, specificity 77.1%) for all grades of hypokalemia with an AUC of 0.773 [0.682 to 0.821]. For grade 1 hypokalemia, the cut-off was 22 mmol (sensitivity: 74.6%, specificity 75.2%, AUC: 0.791 [0.704 to 0.852]), and for grade 2, it was 35 mmol (sensitivity: 70.6%, specificity: 69.2%, AUC: 0.765 [0.612 to 0.871]).

Of 575 patients with hypophosphatemia who received phosphate substitution, 276 (48.0%) did not correct, 249 (43.3%) corrected to normal values, and 50 (8.7%) overcorrected. The median time from the recorded minimum phosphate value to correction, overcorrection, or end of the study period was 15.1 h [8.4 to 24.2]. Multivariable logistic regression analysis showed that for every 10 mmol of phosphate administered, the odds of overcorrection increased by 28% for grade 1 hypophosphatemia (OR 1.33 [1.19 to 1.47], *p* < 0.001) and 26% for grade 2 hypophosphatemia (OR 1.29 [1.15 to 1.43], *p* < 0.001). ROC analysis identified a cut-off for overcorrection of 45 mmol (sensitivity: 69.1%, specificity 75.0%) for all grades of hypophosphatemia with an AUC of 0.753 [0.670 to 0.816]. For grade 1 hypophosphatemia, the cut-off was 29 mmol (sensitivity 66.7%, specificity: 69.0%, AUC: 0.718 [0.588 to 0.822]), and for grade 2, it was 55 mmol (sensitivity: 64.3%, specificity 75.0%, AUC: 0.761 [0.629 to 0.869]).

**Fig. 5:**
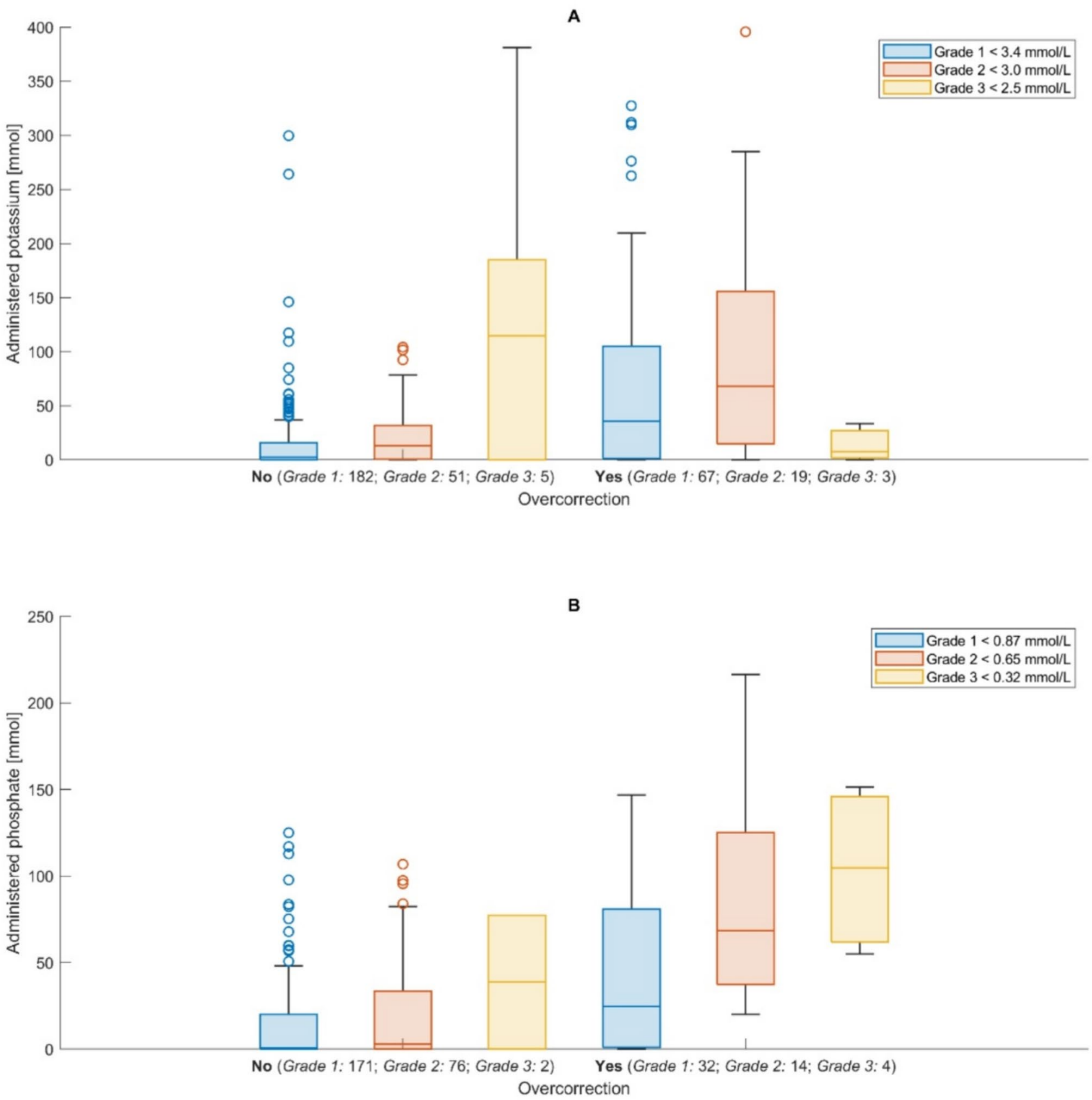
Administered potassium (**A**) and phosphate (**B**) substation [mmol] from the minimum recorded value up to the timepoint when correction or overcorrection occurred, or in case of uncorrected values, the end of the entire study period. The x-axis indicates whether overcorrection occurred and depicts the number of patients in each group. The different colors indicate the grading of the electrolyte disorder.

Figure 5 illustrates the relationship between the administered amounts of potassium and phosphate and the occurrence of overcorrection, stratified by the severity of the initial electrolyte disorder. The results of the regression models are shown in supplementary Tables 3 to 5.

## Discussion

Our study investigated the prevalence and interactions of electrolyte disorders in critically ill patients admitted to a mixed ICU, with an additional focus on identifying safety thresholds for electrolyte replacement. Our findings provide cut-offs that may inform future research and provide safety guidance for electrolyte replacement and avoid overcorrection, suggesting limits of 30 mmol for potassium and 40 mmol for phosphate replacement. Electrolyte abnormalities were highly prevalent on admission and frequently developed throughout the ICU stay, consistent with previous findings in critically ill patients^15,16,33^. In the study population, hyperchloremia was present in 48% of patients on admission and developed in 40% of those with normal admission chloride levels. Neyra and colleagues reported slightly lower incidences of hyperchloremia on admission of approximately 32%^34^. Hyperchloremia has been linked to a higher incidence of renal insufficiency and mortality^11,34,35^. Similar to our data, Stelfox and colleagues found that ICU-acquired hyponatremia and hypernatremia occurred in 11% and 26% of patients during their ICU stay, with significant associations with patient outcomes, including mortality and LOS^5^. Hyperkalemia affected 16% of patients in our dataset, similar to earlier results in a mixed ICU population^29^, and studies reported that abnormal serum potassium levels are linked to increased incidence of arrhythmias, ECG abnormalities and ICU mortality^36,37^. Hypophosphatemia, as well as hyperphosphatemia, were present in approximately 14% of patients on admission. This prevalence aligns with findings from Broman and colleagues, who reported hypophosphatemia and hyperphosphatemia in 13% and 15% of critically ill patients, respectively^38^. Around half of the patients with a normal phosphate level on admission developed phosphate abnormalities during their ICU stay. Hyperphosphatemia is associated with increased mortality, possibly related to renal failure, which reduces phosphate clearance^21,38,39^​.

The literature on overcorrection in electrolyte disorders is limited, but the dangers of aggressive correction protocols are recognized in clinical practice^30,40^. Our data allowed us to define cut-offs around 30 mmol of potassium and 40 mmol of phosphate to avoid overcorrection, with further cut-offs provided for specific grades of phosphate and potassium disorders. Whether overcorrection has a relevant impact on patient outcomes is unknown, but a physiological rationale strongly suggests applying correction only until normal levels are reached. Our data could suggest that 20mmol of potassium for lower grades of hypokalemia and 40mmol for higher grades are reasonable amounts of substitution before another serum level should be checked. Similar cut-offs are provided for phosphate, although substitution and reassessment of serum levels occurred over a larger time span. At the same time, the total amounts needed to correct these electrolytes are highly variable (Fig. 5), probably justifying frequent measurements until the levels have stabilized. These findings underline the importance of personalized electrolyte replacement protocols in critical care.

Electrolytes such as sodium, potassium, calcium, chloride, and phosphate maintain physiological processes, including membrane potential, acid-base balance, and cellular metabolism​^2^. The interplay between different electrolytes is important for physiological homeostasis, and our study provides insights using regression models into how these interactions manifest in critically ill patients. Our findings indicate that electrolyte disorders often coexist. Sodium and chloride are closely linked due to their role in regulating extracellular fluid volume and osmolarity​^34^. In our data, a decrease of 1 mmol/L in bicarbonate predicts an increase of 0.4 mmol/L in chloride, suggesting the development of non-anion gap metabolic acidosis by increasing the chloride-to-bicarbonate ratio. While hyperchloremia was the most prevalent electrolyte disorder in our cohort (56.0% on admission), it showed the lowest correction rate (28.9%) compared to other electrolyte disorders. This finding probably reflects the complex nature of chloride homeostasis, as chloride levels are often a consequence of fluid management strategies and acid-base status rather than a primary therapeutic target. Unlike potassium and phosphate, where aggressive replacement practices are commonly employed for deficiencies due to clear clinical consequences, chloride disorders may require an approach focused on addressing the underlying cause rather than correcting chloride levels. For instance, the management of hyperchloremia often involves adjusting fluid therapy strategies rather than specifically targeting chloride levels^41^.

Potassium plays a vital role in maintaining the resting membrane potential of cells, and its balance is influenced by factors such as acid-base status and cellular turnover^42^. Hypokalemia and hypocalcemia are linked within our dataset, and the risk of arrhythmia may be exacerbated due to their shared involvement in neuromuscular excitability^43^. Our models show that hypokalemia frequently coexists with hypomagnesemia, consistent with the physiological understanding that magnesium is a cofactor for the Na+/K + ATPase pump^44^. This pump helps regulate intracellular potassium levels, and magnesium deficiency can impair its function, leading to persistent hypokalemia and providing arguments to co-administer magnesium with potassium^44^.

Phosphate is crucial for cellular energy metabolism, and its levels are tightly regulated by renal excretion and cellular uptake^1^​. Our data indicate that hypophosphatemia often occurs alongside hypokalemia, suggesting a potential shared mechanism in cellular uptake or renal loss. In contrast, hyperphosphatemia is more likely associated with renal failure and hyperkalemia, as the kidneys are responsible for excreting excess phosphate and other cations^33^​.

Interestingly, our models reveal that certain electrolyte imbalances, such as concurrent hyperchloremia and hypocalcemia, deviate from expected physiological patterns, as hyperchloremic acidosis would be expected to increase calcium levels. This deviation may result from aggressive fluid resuscitation with chloride-rich solutions, leading to dilutional effects and alterations in calcium binding to albumin^27^​. These findings show that therapeutic interventions may impact physiological relationships. Our finding of frequent co-occurrence of electrolyte disorders suggests the need for electrolyte monitoring to capture these interactions and prevent adverse outcomes. It is important to consider that correcting one electrolyte level may cause other potential disorders. Therefore, a physiological understanding of electrolyte balance is needed to interpret measured electrolyte levels. It should allow clinicians to anticipate and manage potential complications arising from these complex interactions and to apply optimal treatment strategies. Our study does not give any recipe for monitoring and administering electrolytes but should call for attention to better understand and inform future studies. Such understanding will hopefully facilitate a personalized approach to the management of electrolyte disturbances. The results of this study have already triggered a local quality improvement process, leading to reduced usage of potassium phosphate solutions when correcting hypokalemia in patients with hyperchloremia, as this practice had resulted in hyperphosphatemia in several patients within the observed study period. However, limited options for IV and enteral potassium preparations restrict the possibility of finding the optimal approach. Additionally, there often is a lack of evidence regarding the best electrolyte substitution thresholds. A recent randomized control trial has challenged the widespread strategy of supplementing potassium to maintain high-normal serum concentrations and prevent atrial fibrillation^45^.

The main strength of our study is the availability of repeated measurements of electrolytes in a large, unselected cohort of ICU patients, allowing, next to epidemiology, an assessment of dynamics and interplay between the electrolytes. Our study has limitations: we only analyzed IV electrolyte replacement, potentially overlooking the effects of oral electrolyte administration. We only observed electrolyte changes over 96 h; therefore, we potentially did not capture all electrolyte disorders developing in the ICU, so electrolyte profiles in long stayers may differ. The retrospective design may have introduced a bias, as the measurements of the electrolytes were not protocolized, and analysis was performed with available data.

Future prospective studies should address these gaps to provide a more comprehensive understanding of electrolyte disorders and their management in the ICU. The low adjusted R^2^ of our regression models indicates poor prediction of changes despite incorporating six dependent variables per model. This suggests that the electrolyte interactions are complex and dependent on many other factors not included in this analysis, such as fluid administration or removal, protein hemostasis including albumin levels, underlying disease, and therapeutic strategies. Our study focuses on the incidence and prevalence of electrolyte disorders in a general ICU population that consists primarily of cardiovascular disorders. Only a minority of the patients in this dataset were admitted for COVID-19. While the underlying diagnosis may explain the electrolyte imbalance and allow the differentiation of the disorder as a consequence of therapy or primary disorder, individual subgroup analyses are beyond the scope of this study. Furthermore, medications commonly impact homeostasis and cause electrolyte imbalances in this patient population^46^. As assessing associations with outcomes was not the aim of this study, we suggest future studies to distinguish between disorders noted on admission and occurring during the ICU stay and between illness-related and iatrogenic disturbances. Our study focused on the first 96 h of ICU stay, which might not capture the full spectrum of electrolyte disorders in long-stay patients. This timeframe was chosen to balance the need for data collection aganst our ICU’s high turn-over rate, given only 22.5% of patients stayed for the full study period. While specific subpopulations like COVID-19 ARDS patients often have longer ICU stays, their relatively small number (2.3%) in our cohort suggests that our findings may remain representative of our general ICU population. Future studies focusing specifically on long-stay patients may reveal different patterns of electrolyte disorders. As we focused on the descriptive approach to show the incidence and prevalence of electrolyte disorders in a large general ICU population, future research should explore potential causal mechanistic relationships for each electrolyte and diagnostic subgroups, including clustering analysis of these groups. We used regression models to assess the simultaneous changes in different electrolyte levels, leading to hypothesis-generating suggestions of potential pathophysiological relationships without any certainty of causation. Optimal electrolyte substitution targets in critically ill patients are unclear and should be assessed along with clinical outcomes. Moreover, we may need different electrolyte reference values in critically ill patients given the high prevalence of these disorders found in this population. Limiting aggressive electrolyte replacement to selected patients should be considered to reduce untoward interactions. Our results demonstrate how changes in one electrolyte level are often associated with alterations in others - for instance, the strong association between potassium and phosphate changes and the inverse relationship between calcium and phosphate. These interactions help explain why standardized replacement protocols may lead to overcorrection in some and under-correction in other patients, emphasizing why safe replacement thresholds must be interpreted within the context of concurrent electrolyte disorders. Similarly, these results indicate that another measurement of phosphate levels may be warranted if potassium levels drop rapidly.

In conclusion, our study provides important descriptive data that may inform us on personalized electrolyte management in critically ill patients. We identified specific thresholds for the most commonly replaced electrolytes, such as potassium and phosphate, that caused overcorrection. Our data suggests that regular monitoring and careful dose titration are essential, given the high prevalence of electrolyte disorders and their complex interactions. Several electrolyte disorders often co-occur, and most changes in electrolyte concentration are frequently interrelated. Future research on developing electrolyte supplementation protocols should consider these interactions, and pooled data may help further refine the cut-offs that guide safe electrolyte replacement.

## Electronic supplementary material

Below is the link to the electronic supplementary material.

Supplementary Material 1

## Data Availability

The datasets used and/or analyzed during the current study are available from the corresponding author upon reasonable request.

## Abbreviations

ICU: Intensive care unit
LOS: Length of stay
IV: Intravenous
SAPS II: Simplified Acute Physiology Score II
SOFA: Sequential organ failure assessment
BUN: Blood urea nitrogen
ROC: Receiver operating characteristic
AUC: Area under the curve
Na^+^: Sodium
K^+^: Potassium
Cl^-^: Chloride
Mg^2+^: Magnesium
PO_4_^3-^: Phosphate
Ca^2+^: Calcium
pO_2_: Partial pressure of oxygen
pCO_2_: Partial pressure of carbon dioxide
GCS: Glasgow Coma Scale
SQL: Structured query language
