# Supplementary material for: Electrolyte disorders in the critically ill: a retrospective analysis
Source: Sci Rep. 2025 Apr 22;15:13943. doi: 10.1038/s41598-025-98677-7 (PMC12015444; doi:10.1038/s41598-025-98677-7)

Online Supplement
Electrolyte disorders in the critically ill: a retrospective analysis

Kaspar F. Bachmann MD^1,2,3^, Benjamin Hess MD^1^, Merli Koitmäe MSc^4,5^, Andreas Bloch MD^1^, Adrian Regli MD PhD^6,7,8^, Annika Reintam Blaser MD PhD^1,2^

1 Department of Intensive Care Medicine, Lucerne Cantonal Hospital, Lucerne, Switzerland

2 Department of Anaesthesiology and Intensive Care, University of Tartu, Tartu, Estonia

3 Department of Intensive Care Medicine, Inselspital, Bern University Hospital, University of Bern, Bern, Switzerland

4 Estonian Genome Center, Institute of Genomics, University of Tartu, Tartu, Estonia

5 Institute of Mathematics and Statistics, University of Tartu, Tartu, Estonia

6 Department of Intensive Care, Fiona Stanley Hospital, Perth, WA, Australia.

7 Medical School, The Notre Dame University, Fremantle, WA, Australia.

8 Medical School, The University of Western Australia, Perth, WA, Australia.

**Corresponding author:**

Kaspar Felix Bachmann

kasparfelix.bachmann@gmail.com

ORCID: 0000-0002-6108-6923

# Supplementary tables

**Supplementary table 1**. The laboratory department at the Lucerne Cantonal Hospital provided the reference values for each electrolyte.

| **Electrolyte** | **Reference Value** |
| --- | --- |
| Sodium | 136 – 145 mmol/L |
| Potassium | 3.4 – 4.5 mmol/L |
| Chloride | 98 – 107 mmol/L |
| Phosphate | 0.87 – 1.45 mmol/L |
| Magnesium | 0.66 – 1.07 mmol/L |
| Ionized Calcium | 1.15 – 1.29 mmol/L |

**Supplementary table 2**. Patient characteristics for the total study population (N=2056) and subgroups based on electrolyte disorders identified on admission. Continuous variables are shown as median [interquartile range] and categorical variables as number (percentage). Percentages in the "N” row are relative to the total population, while other percentages are relative to each column's subgroup. Odds ratios (OR) with 95% confidence intervals were calculated using bivariable logistic regression models, with each electrolyte disorder as the dependent variable. For continuous variables, the OR represents the change in odds of having the specific electrolyte disorder for each one-unit increase in the variable. The OR compares the odds of having the disorder in the given category to the reference category (no disorder) for categorical variables. The ORs in the "No Disorder" column show the odds of not having any electrolyte disorder. ICU: Intensive Care Unit; LOS: Length of Stay; OR: Odds Ratio; SAPS II: Simplified Acute Physiology Score II. Na^+^: Sodium. K^+^: Potassium. Cl^-^: Chloride. Mg^2+^: Magnesium. PO_4_^3-^: Phosphate. Ca^2+^: Calcium.

| **N** | **Total** | **Missing** | **No disorder** | **Hypo Na^+^** | **Hyper Na^+^** | **Hypo K^+^** | **Hyper K^+^** | **Hypo Cl^-^** | **Hyper Cl^-^** | **Hypo Mg^2+^** | **Hyper Mg^2+^** | **Hypo PO_4_^3^_-_** | **Hyper PO_4_^3-^** | **Hypo Ca^2+^** | **Hyper Ca^2+^** |
| --- | --- | --- | --- | --- | --- | --- | --- | --- | --- | --- | --- | --- | --- | --- | --- |
| **Age** | 2056 | 312 (15.2%) | 316 (15.4%) | 268 (13%) | 73 (3.6%) | 170 (8.3%) | 326 (15.9%) | 108 (5.3%) | 977 (47.5%) | 98 (4.8%) | 217 (10.6%) | 293 (14.3%) | 286 (13.9%) | 136 (6.6%) | 39 (1.9%) |
| **Male, n (%)** | 67.1 [55.2 to 76.7] | 65.3 [51.2 to 78.3] | 66.6 [54.4 to 76.8], OR 1 [0.99 to 1], p=0.371 | 68 [56.4 to 77.3], OR 1 [1 to 1.01], p=0.289 | 65.3 [47.8 to 74.7], OR 0.99 [0.97 to 1], p=0.061 | 62.5 [52 to 75.8], OR 0.99 [0.98 to 1], p=0.008 | 70 [59.9 to 77.4], OR 1.01 [1.01 to 1.02], p<0.001 | 67 [57.4 to 75.7], OR 1 [0.99 to 1.02], p=0.544 | 66.6 [54.4 to 76.5], OR 1 [0.99 to 1], p=0.163 | 64.2 [52.4 to 76.6], OR 0.99 [0.98 to 1.01], p=0.374 | 70.6 [60.5 to 78.3], OR 1.02 [1.01 to 1.03], p<0.001 | 68.5 [55.1 to 76.8], OR 1 [0.99 to 1.01], p=0.700 | 70.6 [60.3 to 78.9], OR 1.02 [1.01 to 1.03], p<0.001 | 66.6 [57.7 to 77.6], OR 1.01 [0.99 to 1.02], p=0.327 | 73 [65.7 to 81.6], OR 1.03 [1.01 to 1.05], p=0.016 |
| **Weight, kg** | 1416 (68.9%) | 225 (72.1%) | 226 (71.5%), OR 0.83 [0.63 to 1.09], p=0.173 | 162 (60.4%), OR 1.54 [1.18 to 2], p=0.002 | 51 (69.9%), OR 0.95 [0.57 to 1.58], p=0.848 | 87 (51.2%), OR 2.26 [1.65 to 3.1], p<0.001 | 247 (75.8%), OR 0.66 [0.5 to 0.87], p=0.003 | 60 (55.6%), OR 1.83 [1.24 to 2.71], p=0.002 | 677 (69.3%), OR 0.96 [0.79 to 1.16], p=0.667 | 57 (58.2%), OR 1.61 [1.06 to 2.44], p=0.024 | 155 (71.4%), OR 0.86 [0.63 to 1.17], p=0.328 | 227 (77.5%), OR 0.59 [0.44 to 0.79], p<0.001 | 183 (64%), OR 1.28 [0.98 to 1.67], p=0.066 | 99 (72.8%), OR 0.81 [0.55 to 1.2], p=0.289 | 22 (56.4%), OR 1.72 [0.91 to 3.26], p=0.098 |
| **SAPS II** | 75.1 [66.2 to 86] | 74.1 [69.1 to 88.5] | 77.6 [66.2 to 85.7], OR 1 [1 to 1.01], p=0.436 | 70.2 [62.1 to 81.6], OR 0.98 [0.98 to 0.99], p<0.001 | 74.1 [68.9 to 81.2], OR 0.99 [0.97 to 1], p=0.100 | 71.3 [64.2 to 81.3], OR 0.98 [0.97 to 0.99], p=0.003 | 75.1 [67.7 to 88.8], OR 1.01 [1 to 1.01], p=0.035 | 70 [62.2 to 81], OR 0.98 [0.97 to 0.99], p=0.005 | 75.9 [67.7 to 86.2], OR 1 [1 to 1.01], p=0.678 | 70 [60.7 to 84], OR 0.98 [0.97 to 1], p=0.020 | 77 [66 to 87.4], OR 1 [0.99 to 1.01], p=0.997 | 76.1 [69.1 to 86], OR 1.01 [1 to 1.01], p=0.082 | 76.1 [65.8 to 87.9], OR 1 [1 to 1.01], p=0.464 | 74.1 [67.7 to 87.6], OR 1 [0.99 to 1.01], p=0.847 | 71.1 [62.5 to 80.3], OR 0.99 [0.97 to 1.01], p=0.437 |
| **In hospital mortality, n (%)** | 31 [23 to 42] | 30 [21 to 42.5] | 28 [21 to 36], OR 0.98 [0.97 to 0.99], p<0.001 | 36 [27 to 46], OR 1.02 [1.01 to 1.02], p<0.001 | 39 [25.8 to 51.3], OR 1.02 [1.01 to 1.04], p<0.001 | 34 [25 to 47], OR 1.01 [1 to 1.02], p=0.004 | 38 [29 to 49], OR 1.03 [1.02 to 1.04], p<0.001 | 36 [29.5 to 50], OR 1.02 [1.01 to 1.03], p<0.001 | 30 [22 to 41], OR 0.99 [0.99 to 1], p=0.020 | 32 [24 to 42], OR 1 [0.99 to 1.02], p=0.656 | 34 [26 to 49], OR 1.02 [1.01 to 1.03], p<0.001 | 30 [23 to 41], OR 1 [0.99 to 1.01], p=0.622 | 40 [30 to 52], OR 1.04 [1.03 to 1.04], p<0.001 | 40 [30.5 to 56], OR 1.03 [1.02 to 1.04], p<0.001 | 36 [28.3 to 46.3], OR 1.02 [1 to 1.04], p=0.058 |
| **ICU LOS, days** | 227 (11%) | 50 (16%) | 23 (7.3%), OR 0.65 [0.41 to 1.02], p=0.061 | 40 (14.9%), OR 1.49 [1.03 to 2.16], p=0.033 | 12 (16.4%), OR 1.6 [0.85 to 3.02], p=0.146 | 30 (17.6%), OR 1.87 [1.22 to 2.85], p=0.004 | 53 (16.3%), OR 1.74 [1.24 to 2.43], p=0.001 | 22 (20.4%), OR 2.18 [1.33 to 3.55], p=0.002 | 91 (9.3%), OR 0.7 [0.52 to 0.92], p=0.012 | 9 (9.2%), OR 0.89 [0.44 to 1.79], p=0.741 | 33 (15.2%), OR 1.72 [1.15 to 2.59], p=0.009 | 29 (9.9%), OR 0.96 [0.63 to 1.46], p=0.845 | 56 (19.6%), OR 2.61 [1.85 to 3.68], p<0.001 | 23 (16.9%), OR 1.76 [1.1 to 2.83], p=0.019 | 7 (17.9%), OR 1.82 [0.79 to 4.17], p=0.159 |
| **Hospital LOS, days** | 1 [0.8 to 2] | 0.9 [0.4 to 1.7] | 1 [0.8 to 1.9], OR 0.99 [0.95 to 1.03], p=0.508 | 1.2 [0.8 to 2.5], OR 1.01 [0.98 to 1.05], p=0.474 | 1.2 [0.7 to 3.1], OR 1.04 [0.99 to 1.1], p=0.127 | 1.2 [0.8 to 2.8], OR 1.06 [1.02 to 1.1], p=0.001 | 1.1 [0.8 to 2.3], OR 0.99 [0.95 to 1.02], p=0.469 | 1.4 [0.8 to 2.7], OR 1.02 [0.97 to 1.08], p=0.345 | 1 [0.8 to 2], OR 0.98 [0.95 to 1.01], p=0.154 | 0.9 [0.8 to 1.9], OR 0.97 [0.9 to 1.05], p=0.466 | 1.1 [0.9 to 2.8], OR 1 [0.96 to 1.04], p=0.994 | 1 [0.8 to 2.2], OR 1.01 [0.97 to 1.05], p=0.551 | 1.1 [0.8 to 2.7], OR 1.02 [0.98 to 1.06], p=0.272 | 1.6 [0.9 to 4.3], OR 1.09 [1.05 to 1.13], p<0.001 | 1 [0.7 to 2.4], OR 0.98 [0.88 to 1.1], p=0.766 |
| **Fluids administered, L** | 9 [5 to 15] | 5 [3 to 11] | 9 [5 to 14], OR 0.99 [0.97 to 1], p=0.027 | 9 [5 to 16], OR 1.01 [1 to 1.02], p=0.023 | 7 [2 to 14.3], OR 1 [0.98 to 1.02], p=0.754 | 8.5 [4 to 18], OR 1 [0.99 to 1.02], p=0.545 | 11 [6 to 16], OR 1.01 [1 to 1.02], p=0.069 | 8 [4 to 14.5], OR 0.99 [0.97 to 1.01], p=0.412 | 10 [5 to 15], OR 1 [0.99 to 1.01], p=0.685 | 9 [5 to 16], OR 1 [0.99 to 1.02], p=0.755 | 11 [7 to 16], OR 1 [0.99 to 1.02], p=0.544 | 7 [4 to 15], OR 0.99 [0.98 to 1.01], p=0.293 | 12 [6 to 18], OR 1.01 [1.01 to 1.02], p=0.003 | 12 [6 to 16.5], OR 1.01 [1 to 1.02], p=0.092 | 12 [4.5 to 20.8], OR 1.02 [1 to 1.04], p=0.060 |
| **N** | 1.7 [0.7 to 4.4] | 0.8 [0.3 to 2.7] | 1.4 [0.7 to 3.6], OR 0.98 [0.95 to 1.01], p=0.162 | 2 [0.8 to 4.7], OR 1.01 [0.98 to 1.03], p=0.594 | 2.1 [0.8 to 5.3], OR 1 [0.96 to 1.05], p=0.932 | 1.9 [0.7 to 5.3], OR 1.01 [0.98 to 1.04], p=0.668 | 2.1 [0.8 to 5.1], OR 1.01 [0.99 to 1.04], p=0.214 | 2.3 [0.9 to 4.9], OR 1.02 [0.99 to 1.05], p=0.247 | 1.8 [0.7 to 4.6], OR 1.01 [0.99 to 1.02], p=0.538 | 1.4 [0.7 to 3.9], OR 0.96 [0.91 to 1.02], p=0.170 | 4.1 [1.3 to 7.2], OR 1.07 [1.05 to 1.09], p<0.001 | 1.2 [0.5 to 3.8], OR 0.97 [0.93 to 1], p=0.033 | 2.3 [0.9 to 5.6], OR 1.02 [1 to 1.05], p=0.036 | 3.5 [1.4 to 7.3], OR 1.04 [1.01 to 1.07], p=0.002 | 1.6 [0.5 to 4.6], OR 0.98 [0.9 to 1.06], p=0.549 |
| **Diagnosis** |  |  |  |  |  |  |  |  |  |  |  |  |  |  |  |
| AMI | 308 (15.0%) | 46 (14.7%) | 62 (19.6%) | 22 (8.2%) | 0 (0.0%) | 15 (8.8%) | 33 (10.1%) | 3 (2.8%) | 144 (14.7%) | 14 (14.3%) | 26 (12.0%) | 80 (27.3%) | 17 (5.9%) | 9 (6.6%) | 6 (15.4%) |
| Acid-base, electrolyte disorder | 2 (0.1%) | 2 (0.6%) | 0 (0.0%) | 1 (0.4%) | 0 (0.0%) | 1 (0.6%) | 0 (0.0%) | 1 (0.9%) | 0 (0.0%) | 0 (0.0%) | 0 (0.0%) | 0 (0.0%) | 0 (0.0%) | 0 (0.0%) | 0 (0.0%) |
| Aortic aneurysm | 72 (3.5%) | 8 (2.6%) | 14 (4.4%) | 4 (1.5%) | 3 (4.1%) | 2 (1.2%) | 17 (5.2%) | 1 (0.9%) | 49 (5.0%) | 1 (1.0%) | 10 (4.6%) | 4 (1.4%) | 13 (4.5%) | 8 (5.9%) | 0 (0.0%) |
| Bacterial pneumonia | 35 (1.7%) | 10 (3.2%) | 5 (1.6%) | 11 (4.1%) | 2 (2.7%) | 6 (3.5%) | 2 (0.6%) | 3 (2.8%) | 9 (0.9%) | 1 (1.0%) | 1 (0.5%) | 5 (1.7%) | 2 (0.7%) | 2 (1.5%) | 0 (0.0%) |
| Burn | 1 (0.0%) | 0 (0.0%) | 0 (0.0%) | 0 (0.0%) | 0 (0.0%) | 0 (0.0%) | 0 (0.0%) | 0 (0.0%) | 1 (0.1%) | 0 (0.0%) | 0 (0.0%) | 0 (0.0%) | 0 (0.0%) | 0 (0.0%) | 0 (0.0%) |
| COPD (emphysema/bronchitis) | 17 (0.8%) | 4 (1.3%) | 3 (0.9%) | 2 (0.7%) | 1 (1.4%) | 0 (0.0%) | 6 (1.8%) | 5 (4.6%) | 4 (0.4%) | 1 (1.0%) | 2 (0.9%) | 2 (0.7%) | 4 (1.4%) | 2 (1.5%) | 0 (0.0%) |
| Cardiac arrest | 82 (4.0%) | 19 (6.1%) | 6 (1.9%) | 8 (3.0%) | 2 (2.7%) | 7 (4.1%) | 16 (4.9%) | 3 (2.8%) | 36 (3.7%) | 2 (2.0%) | 19 (8.8%) | 16 (5.5%) | 19 (6.6%) | 14 (10.3%) | 3 (7.7%) |
| Cardiogenic shock | 75 (3.6%) | 7 (2.2%) | 9 (2.8%) | 16 (6.0%) | 3 (4.1%) | 5 (2.9%) | 29 (8.9%) | 10 (9.3%) | 27 (2.8%) | 3 (3.1%) | 6 (2.8%) | 7 (2.4%) | 28 (9.8%) | 9 (6.6%) | 0 (0.0%) |
| Cardiovascular, other | 272 (13.2%) | 18 (5.8%) | 47 (14.9%) | 18 (6.7%) | 10 (13.7%) | 14 (8.2%) | 48 (14.7%) | 3 (2.8%) | 153 (15.7%) | 10 (10.2%) | 76 (35.0%) | 21 (7.2%) | 18 (6.3%) | 16 (11.8%) | 7 (17.9%) |
| Diabetic ketoacidosis | 27 (1.3%) | 3 (1.0%) | 0 (0.0%) | 18 (6.7%) | 3 (4.1%) | 4 (2.4%) | 6 (1.8%) | 18 (16.7%) | 5 (0.5%) | 7 (7.1%) | 6 (2.8%) | 13 (4.4%) | 4 (1.4%) | 5 (3.7%) | 1 (2.6%) |
| Drug overdose | 48 (2.3%) | 18 (5.8%) | 7 (2.2%) | 5 (1.9%) | 6 (8.2%) | 8 (4.7%) | 7 (2.1%) | 4 (3.7%) | 22 (2.3%) | 1 (1.0%) | 1 (0.5%) | 3 (1.0%) | 0 (0.0%) | 3 (2.2%) | 1 (2.6%) |
| GI Neoplasm | 8 (0.4%) | 1 (0.3%) | 0 (0.0%) | 0 (0.0%) | 0 (0.0%) | 0 (0.0%) | 3 (0.9%) | 0 (0.0%) | 8 (0.8%) | 1 (1.0%) | 1 (0.5%) | 0 (0.0%) | 3 (1.0%) | 0 (0.0%) | 0 (0.0%) |
| GI Obstruction | 1 (0.0%) | 0 (0.0%) | 1 (0.3%) | 0 (0.0%) | 0 (0.0%) | 0 (0.0%) | 0 (0.0%) | 0 (0.0%) | 0 (0.0%) | 0 (0.0%) | 0 (0.0%) | 0 (0.0%) | 0 (0.0%) | 0 (0.0%) | 0 (0.0%) |
| GI bleeding | 3 (0.1%) | 0 (0.0%) | 0 (0.0%) | 0 (0.0%) | 0 (0.0%) | 0 (0.0%) | 2 (0.6%) | 0 (0.0%) | 1 (0.1%) | 1 (1.0%) | 0 (0.0%) | 1 (0.3%) | 0 (0.0%) | 1 (0.7%) | 0 (0.0%) |
| Gastrointestinal, other | 123 (6.0%) | 12 (3.8%) | 8 (2.5%) | 17 (6.3%) | 3 (4.1%) | 9 (5.3%) | 23 (7.1%) | 4 (3.7%) | 68 (7.0%) | 11 (11.2%) | 5 (2.3%) | 15 (5.1%) | 37 (12.9%) | 7 (5.1%) | 5 (12.8%) |
| General, other | 43 (2.1%) | 10 (3.2%) | 4 (1.3%) | 2 (0.7%) | 1 (1.4%) | 6 (3.5%) | 8 (2.5%) | 1 (0.9%) | 26 (2.7%) | 0 (0.0%) | 3 (1.4%) | 3 (1.0%) | 7 (2.4%) | 2 (1.5%) | 0 (0.0%) |
| Hematologic, other | 6 (0.3%) | 1 (0.3%) | 1 (0.3%) | 0 (0.0%) | 0 (0.0%) | 0 (0.0%) | 2 (0.6%) | 0 (0.0%) | 4 (0.4%) | 0 (0.0%) | 1 (0.5%) | 0 (0.0%) | 1 (0.3%) | 0 (0.0%) | 0 (0.0%) |
| Hepatic failure | 23 (1.1%) | 2 (0.6%) | 2 (0.6%) | 5 (1.9%) | 1 (1.4%) | 3 (1.8%) | 8 (2.5%) | 4 (3.7%) | 11 (1.1%) | 4 (4.1%) | 4 (1.8%) | 2 (0.7%) | 6 (2.1%) | 2 (1.5%) | 0 (0.0%) |
| Intracerebral hemorrhage | 68 (3.3%) | 14 (4.5%) | 16 (5.1%) | 10 (3.7%) | 1 (1.4%) | 10 (5.9%) | 5 (1.5%) | 4 (3.7%) | 21 (2.1%) | 1 (1.0%) | 3 (1.4%) | 8 (2.7%) | 7 (2.4%) | 3 (2.2%) | 1 (2.6%) |
| Multiple trauma (excluding head trauma) | 76 (3.7%) | 13 (4.2%) | 15 (4.7%) | 5 (1.9%) | 2 (2.7%) | 4 (2.4%) | 11 (3.4%) | 2 (1.9%) | 37 (3.8%) | 1 (1.0%) | 1 (0.5%) | 6 (2.0%) | 9 (3.1%) | 9 (6.6%) | 1 (2.6%) |
| Neurologic infection | 9 (0.4%) | 0 (0.0%) | 2 (0.6%) | 1 (0.4%) | 0 (0.0%) | 2 (1.2%) | 1 (0.3%) | 0 (0.0%) | 4 (0.4%) | 0 (0.0%) | 0 (0.0%) | 4 (1.4%) | 0 (0.0%) | 0 (0.0%) | 0 (0.0%) |
| Neurologic neoplasm | 10 (0.5%) | 2 (0.6%) | 2 (0.6%) | 0 (0.0%) | 0 (0.0%) | 1 (0.6%) | 0 (0.0%) | 0 (0.0%) | 7 (0.7%) | 0 (0.0%) | 2 (0.9%) | 1 (0.3%) | 1 (0.3%) | 0 (0.0%) | 0 (0.0%) |
| Neurologic, other | 131 (6.4%) | 12 (3.8%) | 21 (6.6%) | 18 (6.7%) | 5 (6.8%) | 7 (4.1%) | 12 (3.7%) | 5 (4.6%) | 64 (6.6%) | 3 (3.1%) | 11 (5.1%) | 11 (3.8%) | 18 (6.3%) | 3 (2.2%) | 2 (5.1%) |
| Neuromuscular disease | 3 (0.1%) | 2 (0.6%) | 0 (0.0%) | 0 (0.0%) | 0 (0.0%) | 1 (0.6%) | 0 (0.0%) | 0 (0.0%) | 1 (0.1%) | 0 (0.0%) | 0 (0.0%) | 1 (0.3%) | 0 (0.0%) | 0 (0.0%) | 0 (0.0%) |
| Pancreatitis | 5 (0.2%) | 1 (0.3%) | 1 (0.3%) | 0 (0.0%) | 0 (0.0%) | 0 (0.0%) | 1 (0.3%) | 0 (0.0%) | 3 (0.3%) | 0 (0.0%) | 0 (0.0%) | 1 (0.3%) | 0 (0.0%) | 2 (1.5%) | 1 (2.6%) |
| Pulmonary edema (noncardiac) | 64 (3.1%) | 15 (4.8%) | 4 (1.3%) | 16 (6.0%) | 4 (5.5%) | 6 (3.5%) | 5 (1.5%) | 5 (4.6%) | 28 (2.9%) | 1 (1.0%) | 5 (2.3%) | 15 (5.1%) | 9 (3.1%) | 8 (5.9%) | 1 (2.6%) |
| Pulmonary embolism | 14 (0.7%) | 2 (0.6%) | 3 (0.9%) | 3 (1.1%) | 0 (0.0%) | 3 (1.8%) | 1 (0.3%) | 1 (0.9%) | 5 (0.5%) | 0 (0.0%) | 2 (0.9%) | 1 (0.3%) | 1 (0.3%) | 1 (0.7%) | 0 (0.0%) |
| Renal, other | 41 (2.0%) | 2 (0.6%) | 1 (0.3%) | 7 (2.6%) | 3 (4.1%) | 2 (1.2%) | 9 (2.8%) | 1 (0.9%) | 28 (2.9%) | 6 (6.1%) | 1 (0.5%) | 0 (0.0%) | 18 (6.3%) | 3 (2.2%) | 1 (2.6%) |
| Respiratory Disease, other | 48 (2.3%) | 7 (2.2%) | 7 (2.2%) | 4 (1.5%) | 2 (2.7%) | 3 (1.8%) | 9 (2.8%) | 2 (1.9%) | 21 (2.1%) | 1 (1.0%) | 2 (0.9%) | 6 (2.0%) | 11 (3.8%) | 2 (1.5%) | 1 (2.6%) |
| Respiratory cancer | 33 (1.6%) | 3 (1.0%) | 9 (2.8%) | 4 (1.5%) | 0 (0.0%) | 1 (0.6%) | 6 (1.8%) | 2 (1.9%) | 10 (1.0%) | 2 (2.0%) | 2 (0.9%) | 0 (0.0%) | 7 (2.4%) | 0 (0.0%) | 0 (0.0%) |
| Rhythm disturbance | 51 (2.5%) | 16 (5.1%) | 7 (2.2%) | 3 (1.1%) | 0 (0.0%) | 2 (1.2%) | 5 (1.5%) | 2 (1.9%) | 22 (2.3%) | 3 (3.1%) | 6 (2.8%) | 12 (4.1%) | 6 (2.1%) | 3 (2.2%) | 0 (0.0%) |
| Seizures (no structural disease) | 35 (1.7%) | 6 (1.9%) | 6 (1.9%) | 7 (2.6%) | 3 (4.1%) | 7 (4.1%) | 4 (1.2%) | 5 (4.6%) | 12 (1.2%) | 2 (2.0%) | 2 (0.9%) | 5 (1.7%) | 3 (1.0%) | 3 (2.2%) | 2 (5.1%) |
| Sepsis | 76 (3.7%) | 11 (3.5%) | 1 (0.3%) | 35 (13.1%) | 2 (2.7%) | 16 (9.4%) | 18 (5.5%) | 11 (10.2%) | 29 (3.0%) | 6 (6.1%) | 9 (4.1%) | 16 (5.5%) | 20 (7.0%) | 8 (5.9%) | 3 (7.7%) |
| Stroke | 29 (1.4%) | 4 (1.3%) | 6 (1.9%) | 1 (0.4%) | 3 (4.1%) | 3 (1.8%) | 3 (0.9%) | 0 (0.0%) | 15 (1.5%) | 1 (1.0%) | 3 (1.4%) | 5 (1.7%) | 6 (2.1%) | 0 (0.0%) | 0 (0.0%) |
| Subarachnoid hemorrhage, intracranial aneurysm | 34 (1.7%) | 5 (1.6%) | 8 (2.5%) | 2 (0.7%) | 0 (0.0%) | 6 (3.5%) | 0 (0.0%) | 2 (1.9%) | 18 (1.8%) | 2 (2.0%) | 0 (0.0%) | 7 (2.4%) | 0 (0.0%) | 1 (0.7%) | 0 (0.0%) |
| Trauma involving the head | 92 (4.5%) | 24 (7.7%) | 20 (6.3%) | 6 (2.2%) | 10 (13.7%) | 10 (5.9%) | 10 (3.1%) | 0 (0.0%) | 39 (4.0%) | 6 (6.1%) | 1 (0.5%) | 11 (3.8%) | 5 (1.7%) | 3 (2.2%) | 1 (2.6%) |
| Trauma, other | 23 (1.1%) | 5 (1.6%) | 5 (1.6%) | 2 (0.7%) | 3 (4.1%) | 1 (0.6%) | 3 (0.9%) | 2 (1.9%) | 11 (1.1%) | 0 (0.0%) | 2 (0.9%) | 2 (0.7%) | 1 (0.3%) | 2 (1.5%) | 0 (0.0%) |
| AMI | 308 (15.0%) | 46 (14.7%) | 62 (19.6%) | 22 (8.2%) | 0 (0.0%) | 15 (8.8%) | 33 (10.1%) | 3 (2.8%) | 144 (14.7%) | 14 (14.3%) | 26 (12.0%) | 80 (27.3%) | 17 (5.9%) | 9 (6.6%) | 6 (15.4%) |

**Supplementary table 3.** A multivariable regression model was used to assess the risk of overcorrection in hypokalemic patients. Odds ratios with 95% confidence intervals and p-values were calculated to determine the association between a potassium dose of 10 mmol and the odds of overcorrection, stratified by the severity grades of hypokalemia. Hypokalemia grades were defined as grade 1: < 3.4 and ≥ 3, grade 2: < 3 and ≥ 2.5, grade 3: < 2.5.

| **Variable** | **OR, increase per 10 mmol of infused potassium** | **pValue** |
| --- | --- | --- |
| Hypokalemia grade 1 * infused 10 mmol of potassium | 1.19 [1.11 to 1.28] | < 0.001 |
| Hypokalemia grade 2 * infused 10 mmol of potassium | 1.18 [1.07 to 1.30] | < 0.001 |
| Hypokalemia grade 3 * infused 10 mmol of potassium | 0.95 [0.75 to 1.21] | 0.677 |

**Supplementary table 4.** A multivariable regression model was applied to evaluate the risk of overcorrection in hypophosphatemic patients. Odds ratios, along with 95% confidence intervals and p-values, were calculated to assess the relationship between a 10 mmol phosphate dose and the odds of overcorrection, stratified by the severity grades of hypophosphatemia. Hypophosphatemia grades were defined as grade 1: < 0.87 and ≥ 0.65, grade 2: < 0.65 and ≥ 0.32, grade 3: < 0.32 and

| **Variable** | **OR, increase per 10 mmol of infused phosphate** | **pValue** |
| --- | --- | --- |
| Hypophosphatemia grade 1 * infused 10 mmol of phosphate | 1.33 [1.19 to 1.47] | < 0.001 |
| Hypophosphatemia grade 2 * infused 10 mmol of phosphate | 1.29 [1.15 to 1.43] | < 0.001 |
| Hypophosphatemia grade 3 * infused 10 mmol of phosphate | 1.58 [1.14 to 2.20] | 0.006 |

# Supplementary Figures

**Supplementary Figure 1**. Representation of the chosen measurement intervals to assess the interplay between electrolytes, using visual representation from data of one patient with four eligible measurement intervals. The green and red areas indicate the electrolytes set used for the start and end of an interval, respectively. The time differences within each set are given in the figure. With multiple intervals, some electrolyte sets are used as start of the previous and end point of the next interval. The smaller dots represent additional measurements (point of care) not used in the interplay analysis, as they are not part of full sets of electrolytes. iCA: ionized Calcium. PO4: Phosphate. Mg: Magnesium. Na: Sodium. K: Potassium. Cl: Chloride.

**
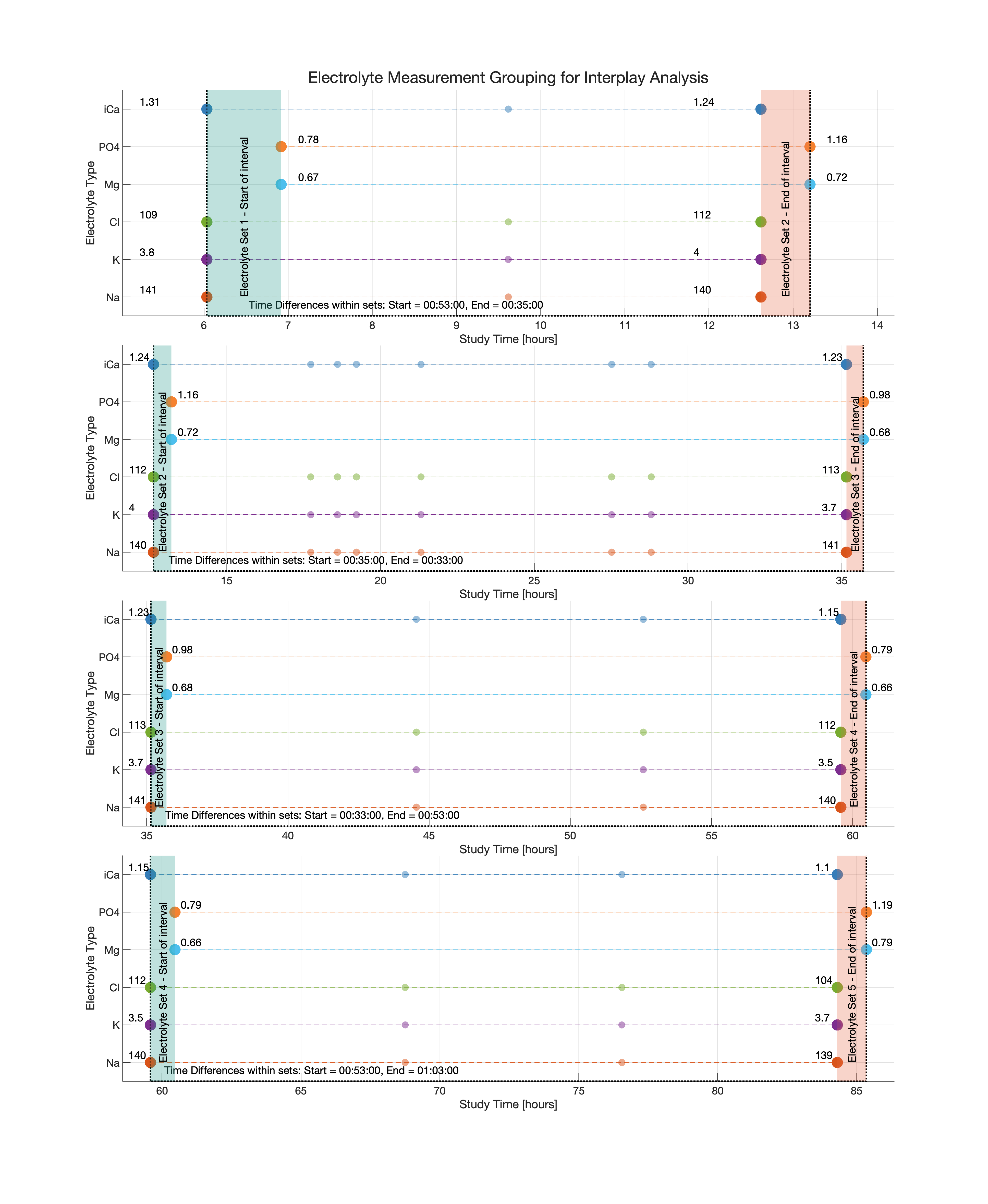
**

**Supplementary Figure 2**. The heatmap illustrates the temporal trends of electrolyte disorders (A to F) by displaying the frequency of each disorder and the corresponding number of patients per day."

#
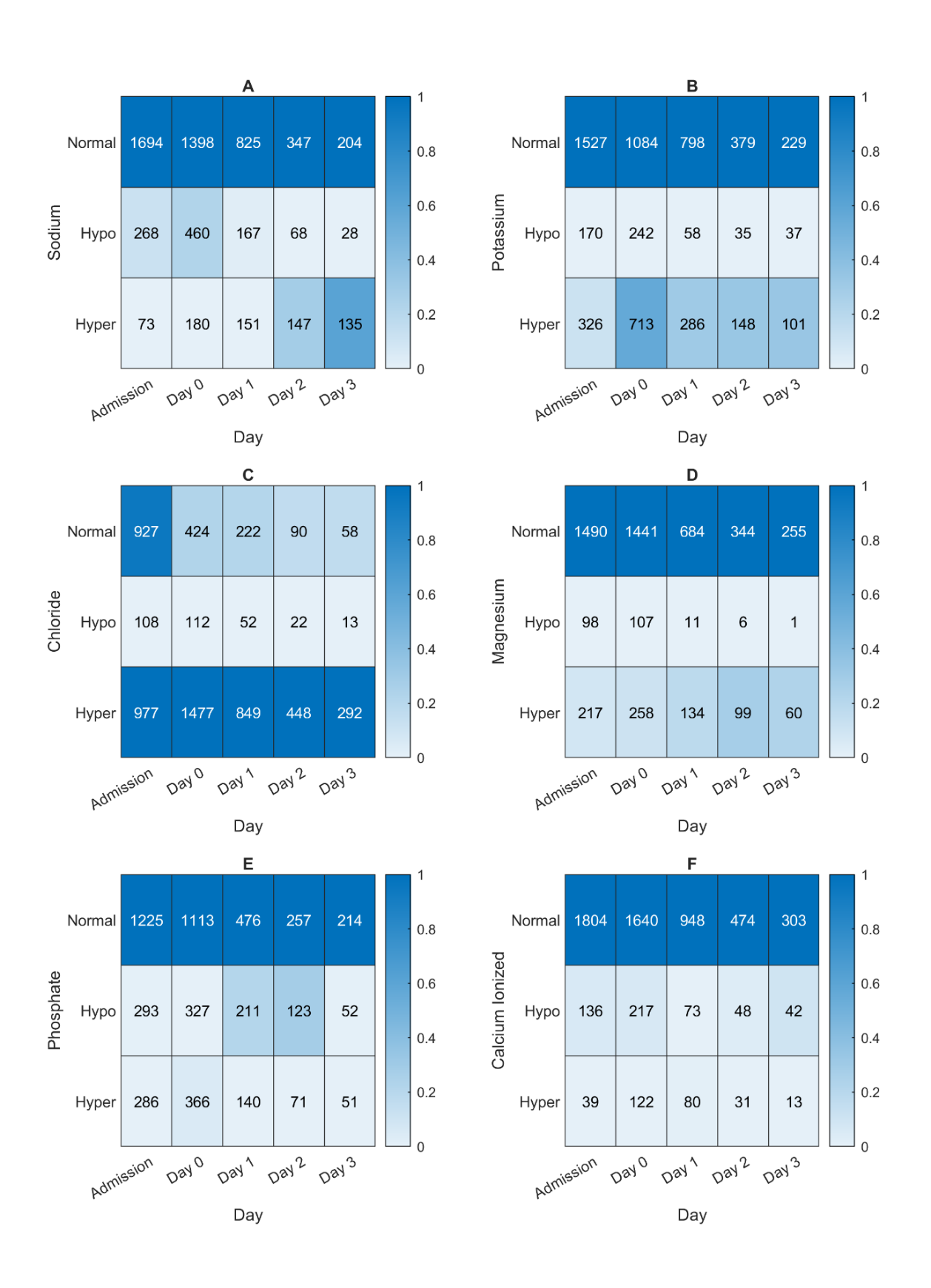

Supplement: Supplementary file 1 — Supplementary Material 1 [file 41598_2025_98677_MOESM1_ESM.docx]
